# Co-option of the limb patterning program in cephalopod eye development

**DOI:** 10.1186/s12915-021-01182-2

**Published:** 2022-01-05

**Authors:** Stephanie Neal, Kyle J. McCulloch, Francesca R. Napoli, Christina M. Daly, James H. Coleman, Kristen M. Koenig

**Affiliations:** 1 John Harvard Distinguished Science Fellowship Program, Harvard University, Cambridge, MA 02138 USA; 2 Department of Organismic and Evolutionary Biology, Harvard University, Cambridge, MA 02138 USA

**Keywords:** Eye, Cephalopod, Eye evolution, Lens, Vision, Spiralia, Wnt, Limb patterning, Dlx

## Abstract

**Background:**

Across the Metazoa, similar genetic programs are found in the development of analogous, independently evolved, morphological features. The functional significance of this reuse and the underlying mechanisms of co-option remain unclear. Cephalopods have evolved a highly acute visual system with a cup-shaped retina and a novel refractive lens in the anterior, important for a number of sophisticated behaviors including predation, mating, and camouflage. Almost nothing is known about the molecular-genetics of lens development in the cephalopod.

**Results:**

Here we identify the co-option of the canonical bilaterian limb patterning program during cephalopod lens development, a functionally unrelated structure. We show radial expression of transcription factors *SP6-9/sp1*, *Dlx/dll*, *Pbx/exd*, *Meis/hth*, and a *Prdl* homolog in the squid *Doryteuthis pealeii*, similar to expression required in *Drosophila* limb development. We assess the role of Wnt signaling in the cephalopod lens, a positive regulator in the developing *Drosophila* limb, and find the regulatory relationship reversed, with ectopic Wnt signaling leading to lens loss.

**Conclusion:**

This regulatory divergence suggests that duplication of SP6-9 in cephalopods may mediate the co-option of the limb patterning program. Thus, our study suggests that this program could perform a more universal developmental function in radial patterning and highlights how canonical genetic programs are repurposed in novel structures.

**Supplementary Information:**

The online version contains supplementary material available at 10.1186/s12915-021-01182-2.

## Main text

### Background

In the Metazoa, homologous networks of transcription factors are necessary for the development of some analogous structures in distantly related taxa. The limb patterning program is an example of this developmental process homology [1–3]. The limb program was first identified in the development of the proximal-distal axis of the *Drosophila* leg*.* The transcription factor *SP6-9/sp1* is upstream of other program members, *Dlx/dll*, *Pbx/exd*, *Meis/hth*, *Dac*, and *Arx/ar*, each required for patterning specific regions of limb outgrowth [3–9]. This network is necessary in both vertebrate and cephalopod limb development and is expressed in a similar proximodistal pattern in a diversity of outgrowths [1, 3, 5, 10–25]. This suggests that, although each appendage is not homologous, an outgrowth program may have been present in the ancestor. Current fossil evidence and the prevalence of limbless taxa do not support an ancestor with appendages and therefore the network’s ancestral function remains unclear [1–3]. Many alternative hypotheses have been proposed, including an ancestral role in the nervous system, body axis formation, and radial patterning [2, 3, 26–30]. To understand the nature of this homology and how these co-option events occur, experiments with better sampling across the phylogeny of animals and greater diversity of developmental context are required.

Recent work identified a duplication of SP6-9 in cephalopods [31]. Both paralogs are expressed in the developing limb in the squid *Doryteuthis pealeii*, while one paralog, *DpSP6-9a*, shows unique expression in the lens-making cells during eye development [31]. With SP6-9 a known regulator in the limb patterning program, this new domain of expression could result in the co-option of the program in the cephalopod eye, providing a useful heterologous developmental context to better understand the network’s function.

The image-forming eye is a classic example of biological complexity and the lens is a requisite innovation in all high-resolution visual systems [32–38]. Cephalopods have a single-chambered eye, morphologically convergent with the vertebrate eye, composed of a cup-shaped retina and a single refractive lens [39]. Here we perform the first in-depth molecular description of lens development in the squid *Doryteuthis pealeii*, we identify spatiotemporal expression of the limb patterning program in the developing eye and lens, and we demonstrate a negative regulatory role of canonical Wnt signaling upstream of the program.

## Results and discussion

### Cephalopod lentigenic cell differentiation and early anterior segment heterogeneity

**Fig. 1:**
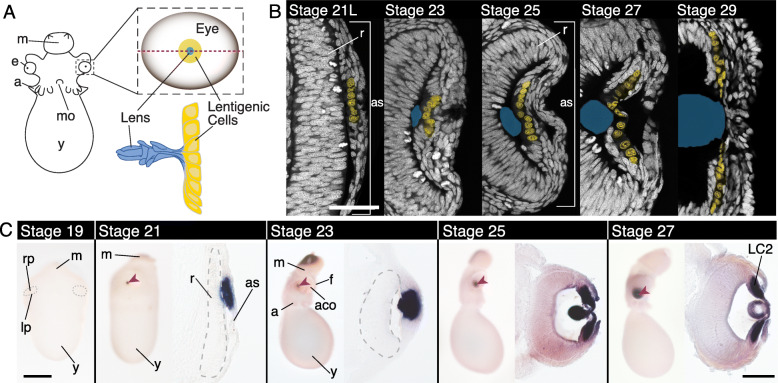
Lentigenic cell differentiation and *DpS-Crystallin* expression in the squid. **A** Cartoon diagram of a squid embryo (anterior), en face cartoon of the developing eye (red dotted line shows cross-section plane throughout the paper), and developing lentigenic cells and lens (cartoon of lens and lentigenic cells based on [43]). **B** Cross-section of the developing anterior segment at Arnold stages 21 late, 23, 25, 27, and 29 identifying differentiation of lentigenic cells [49]. White: Sytox-Green labeling nuclei, Yellow: False-colored lentigenic cell nuclei corresponding to the LC2 population identified by nuclear morphology [43, 44, 46]. Blue is the outline of the lens, as identified using phalloidin staining (not shown). First evidence of LC2 cells is late stage 21. Lentigenic cell number multiplies and distribution grows across the anterior segment (*as)* throughout development. Scale is 50 μm. **C** In situ hybridization of *DpS-Crystallin* in whole-mount and cryo-section. Stage 19 is an anterior view, the boundary between the retina placode and the lip cells is highlighted with a dotted line. No *DpS-Crystallin* expression is apparent at this stage. Stages 21–27 are shown in a lateral view of the embryo on the left and a cross-section of the eye on the right. Anterior of the embryo is down in the sections. The retina is outlined with a dashed grey line in stage 21 and 23. *DpS-Crystallin* expression corresponds with LC2 population. Scale is 500 μm in whole mount images. Scale is 100 μm in sectioned images. *as*, anterior segment; *a*, arm; *aco*, anterior chamber organ; *e*, eye; *f*, funnel; LC2, lentigenic cell population; *lp*, lip; *m*, mantle; *mo*, mouth; *rp*, retina placode; *r*, retina; *y*, yolk. Red arrow highlights the lens

**Fig. 2:**
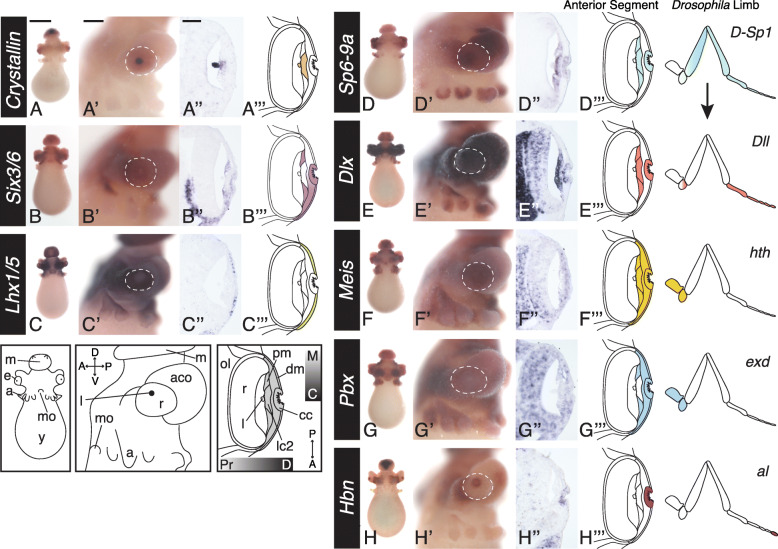
Limb patterning program expressed in the developing anterior segment. For each gene: left to right, anterior whole-mount view, lateral whole-mount view (anterior left), cross-section (anterior is down), cartoon summary is specific to only the anterior segment expression, excluding brain and retina expression for clarity. Dotted white outline in lateral view outlines the perimeter of the eye. **A**–**C** Defining cell populations in the developing anterior segment at stage 23. **A**, **A’**, **A”** *DpS-Crystallin* expression in the anterior segment at stage 23, expressed in the proximal, central cells corresponding with the LC2 cells (*lc2*). Expression is also apparent in the lens. **B**, **B’**, **B”** Expression of *DpSix3/6.* **B”** Expression is apparent in the distal, central cup cells (*cc*) and the proximal-marginal (*pm*) anterior segment cells. The proximal-central cells (*lc2*) lack expression of *DpSix3/6*. **C**, **C’**, **C”** *DpLhx1/5* expression. **C”** Expression of *DpLhx1/5* is found in the distal-marginal cell (*dm*) population. Expression is excluded from the central cup (*cc*). **D**–**G** Expression of the limb patterning program genes. Summary of the proximodistal expression of each *Drosophila* homolog during proximodistal patterning of the limb is shown on the right. **H** Prd-like homolog *Homeobrain* (*Hbn*) expression in the distal, central cup cells. *a*, arms; *aco*, anterior chamber organ; *cc*, cup cells; *dm,* distal-marginal cells; *e*, eye; *l*, lens; *lc2,* LC2 cells; *m*, mantle; *mo*, mouth; *pm,* proximal-marginal cells; r, retina; *y*, yolk. Anterior segment highlighted in grey in the cartoon. Orientation abbreviations: M, marginal; C, central; Pr, proximal; D, Distal; A, anterior; P, posterior. Scale for whole-mount anterior view is 500 μm. Scale for lateral whole-mount view 200 μm. Scale for sectioned images 50 μm

The anterior of the cephalopod eye, or the anterior segment, is composed primarily of lens generating cells (lentigenic cells) [40–42]. Lentigenic cells are arranged circumferentially around the developing lens and extend long cellular processes, fusing into plates to form the lens (Fig. 1A) [40, 41, 43–45]. We identified the first evidence of differentiated lentigenic cells starting at late stage 21, using a previously described nuclear morphology, unique to one of the three lentigenic cell types (LC2) (Figs. 1B and 2A) [43, 44, 46]. The number of LC2 cells continues to grow until reaching pre-hatching stage (stage 29). We performed staged in situ hybridization for a homolog of *DpS-Crystallin*, the most abundant family of proteins in the cephalopod lens [47, 48] (Supplemental Figure 1). The first evidence of expression correlates with changes in nuclear morphology at stage 21 (Fig. 1C).

We sought to understand the molecular heterogeneity of cells in the early developing anterior segment, of which nothing is currently known. Using previously published candidates and RNA-seq data, we performed in situ hybridization screens at stage 23 to identify unique cell populations [46, 50]. We find *DpSix3/6* at stage 23 expressed in the anterior segment in the distal cells that make a central cup (*cc*), as well as a marginal population of cells in the most proximal tissue (*pm*) (Fig. 2B”, Supplemental Figure 2, Supplemental Figure 3). The proximal central cells lacking *DpSix3/6* expression correspond to the LC2 population (Fig. 2A”, B”). Asymmetry along the animal anterior-posterior axis in the eye is also apparent, with enrichment on the anterior side of the animal (Fig. 2B”). We also find the gene *DpLhx1/5*, expressed in a distal-marginal population of cells in the anterior segment (*dm*), and excluded from the distal central cup cells (*cc*) (Fig. 2C”, Supplemental Figure 2, Supplemental Figure 3). Together these genes show distinct populations of cells present early in development and provide a helpful molecular map of the anterior segment tissue at this time point: central cup cells (*cc*), LC2 cells (*lc2*), proximal-marginal cells (*pm*), and distal-marginal cells (*dm*) (Fig. 2).

### Proximal-distal limb patterning genes in the anterior segment of the cephalopod

To assess whether genes involved in appendage patterning may be required for cephalopod lens development, we identified and performed in situ hybridization for the genes *Dlx*, *Meis*, *Pbx*, and *Dac* at stages 21 and 23 (Fig. 2, Supplemental Figure 2, Supplemental Figure 3)*.* All genes were clearly expressed in the developing anterior segment and lentigenic cells with the exception of *DpDac* (Fig. 2E–G, Supplemental Figure 2C-2J’, Supplemental Figure 3). We find *DpSP6-9a* and *DpDlx* have overlapping expression, in the central cup cells (*cc*) and all proximal cells (LC2 and *pm*) (Fig. 2D–E”, Supplemental Figure 3)*. DpMeis* and *DpPbx* are both broadly expressed in the anterior segment during lens development, with *DpPbx* excluded from the LC2 cells (Fig. 2F”, G”, Supplemental Figure 3).

It is known that the transcription factor *aristaless* is necessary for the most distal tip of the *Drosophila* limb in the limb program [9]. The evolutionary relationship of Prd-like homologs (Arx/Aristaless, Alx/Aristaless-like, Rx/Retinal Homeobox, and Hbn/Homeobrain) is ambiguous across species [51]. We identified three candidate Prd-like genes in *D. pealeii* and performed in situ hybridization for all three homologs, *DpHbn*, *DpPrdl-1*, and *DpPrdl-2* (Supplemental Figure 2K, L) [46]. *DpHbn* is expressed in the anterior segment in the distal central cup cells (*cc*) while *DpPrdl-1* and *DpPrdl-2* are excluded from the eye (Fig. 2H” and Supplemental Figure 2C, C’, K and L, Supplemental Figure 3). *DpHbn*’s central, distal expression recapitulates *aristaless* expression in the developing *Drosophila* limb.

Our data show that the majority of the proximodistal patterning genes in the developing limb, including *SP6-9*, *Dlx*, *Meis*, *Pbx*, as well as the Prd-like homolog, *Hbn*, show expression in concentric and overlapping cell populations surrounding the developing lens in the squid (Fig. 2). This pattern of expression is similar to the bullseye-like pattern of expression of these genes in the developing *Drosophila* limb imaginal disc and suggests a co-option of this regulatory program for a new function: patterning the cephalopod anterior segment and lens [14].

### Canonical Wnt signaling genes expressed during anterior segment development

**Fig. 3:**
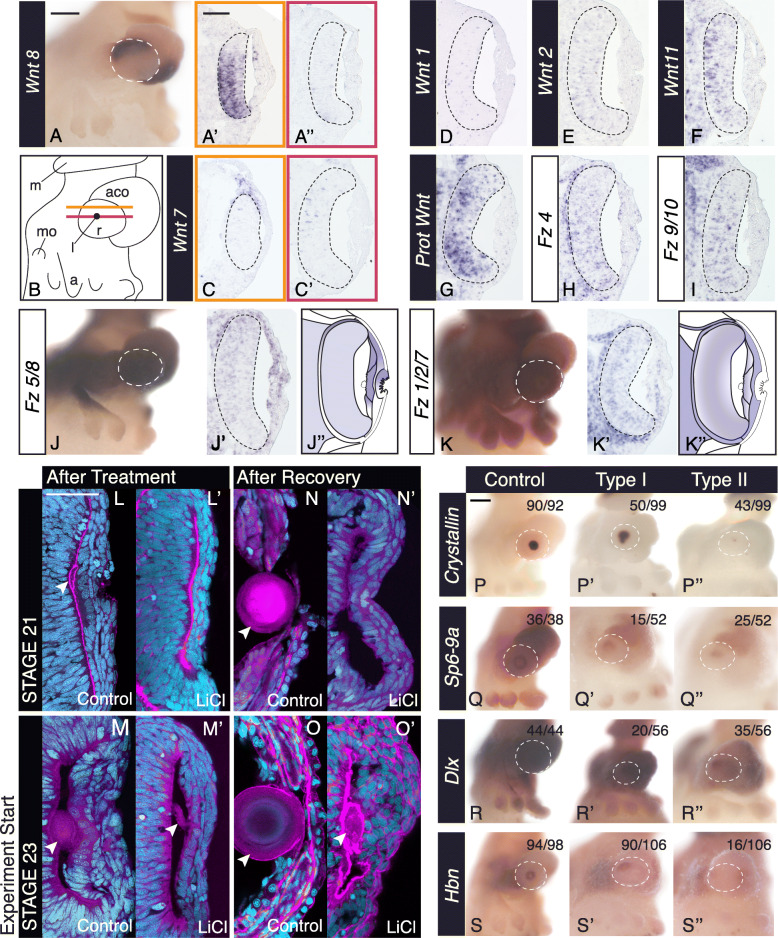
Wnt signaling pathway expression in the developing squid eye. **A**–**G** *Wnt* gene expression at stage 23. Based on expression, Wnt7, Wnt8, Wnt2, Wnt11, and Prot Wnt are possible candidates to signal the anterior segment. **A** Lateral, whole-mount expression of *Wnt8*. **A’** Dorsal retina expression of *Wnt8*. Location of the section indicated by the orange line in **B**. **A”** Central section lacking retina expression. Location of the section indicated by the red line in **B**. **B** Cartoon of the lateral whole-mount embryo at stage 23. Orange and red lines correspond to the location of the two sections shown in **A**, **A’**, and **C**, **C’**. **D**–**G** Expression of other *Wnt* homologs in central sections. **H**–**K** Expression of Frizzled receptors at stage 23. *Fz1/2/7* shows asymmetric expression and *Fz5/8* shows specific exclusion from the central cup cells. **J** and **K** are lateral view of the whole mount expression. **J”** and **K”** are cartoons of expression in **J’** and **K’** respectively. Gradients of expression show intracellular asymmetries in the anterior segment. Black dotted line in sectioned images shows the perimeter of the retina. **L**–**O** Anterior segment and lens morphology after Wnt agonist treatment (LiCl). Embryos were cryosectioned and stained with Sytox-green (nuclei, cyan) and phalloidin (F-actin, magenta). **L** and **L’** Control and LiCl agonist treatments started at stage 21, treated for 24 h and fixed immediately. **M** and **M’** Control and Wnt agonist (LiCl) treatments started at stage 23 for 24 h and fixed immediately. **N** and **N’** Control and Wnt agonist (LiCl) treatments started at stage 21, treated for 24 h and allowed to recover for 48 h and fixed. **O** and **O’** Control and Wnt agonist (LiCl) treatments started at stage 23, treated for 24 h and allowed to recover for 48 h and fixed. Arrowhead highlights the lens. **P**–**S** In situ hybridization of anterior segment markers after 24-h control and LiCl treatments starting at stage 23. Phenotypes are characterized as Type I (mild) and Type II (severe). The white dotted line outlines the eye in the lateral image. The number of eyes scored in control and the two phenotypes is found in LiCl-treated animals in the top right corner. Scale for all lateral whole-mount view images is 200 μm. Scale for all sectioned images is 50 μm. Anterior is down in all sectioned images. White dotted line in whole mount images identifies the perimeter of the eye. *m*, mantle; *a*, arms; *aco*, anterior chamber organ; *mo*, mouth; *r*, retina; *l*, lens

The duplication of SP6-9 in cephalopods may provide a substrate for the evolution of *cis*-regulation, resulting in novel expression of the limb patterning program in the cephalopod lens. In *Drosophila* appendage outgrowth, active Wnt signaling is upstream of the expression of SP6-9 [52, 53]. To assess whether Wnt may be acting upstream in the cephalopod anterior segment or whether novel regulatory mechanisms may be at play, we performed in situ hybridization for members of the Wnt signaling pathway at stage 21 and stage 23 (Fig. 3, Supplemental Figure 4). We were interested in identifying cells in the anterior segment or in adjacent tissue that may be a source of the Wnt morphogen. We performed in situ hybridization for seven Wnt homologs, with most *Wnt* genes expressed in the retina (Fig. 3A’, C’, and D–G). *DpWnt8*, *DpWnt11*, and *DpProtostome-specific Wnt* show the most robust retinal expression (Fig. 3A’, F, and G), and *DpWnt7* is the only Wnt expressed in the anterior segment (Fig. 3C). *DpWnt6* showed no evidence of expression in the developing eye (data not shown). These data support the hypothesis that Wnt signals emanating from the anterior segment or neighboring tissues could regulate anterior segment development.

To identify cells with potential active Wnt signaling, we analyzed the expression of Fz genes, which encode a family of Wnt receptors. We find that *DpFz* receptors are expressed broadly throughout the embryo. A subset of these (e.g. *DpFz1/2/7*, *DpFz4*, and *DpFz5/8*) are expressed in a subset of cells in the anterior segment, while others, like *DpFz9/10*, are excluded from the anterior segment (Fig. 3H–K, Supplemental Figure 4). On close examination, we find that *DpFz5/8* is excluded asymmetrically in the anterior segment and may be important for anterior-posterior patterning (Fig. 3J’, J”, Supplemental Figure 4D). *DpFz1/2/7* is excluded from the distal-marginal cells and central cup cells and interestingly, the central cup cells lacking *DpFz1/2/7* are those that express all the limb patterning program genes (Fig. 3K’, K”, Supplemental Figure 4D). These data suggested active Wnt signaling may be important in the cephalopod anterior segment.

### Ectopic Wnt activation leads to the loss of the lens

To assess the hypothesis that Wnt signaling is playing a regulatory role in anterior segment development, we utilized well-characterized pharmacological compounds that act as agonists and antagonists of the Wnt pathway [54–57]. We empirically determined a working concentration of LiCl (0.15 M), CHIR99021 (250 μm), and Quercetin (50 μM). We bathed embryos in the compound or vehicle control for 24 h at stage 21, the onset of lentigenic cell differentiation, and immediately fixed thereafter. Embryos were sectioned and assessed for phenotypes. Stage 21 control embryos show a thickened anterior segment, identifiable lentigenic cells, and small lens primordia (Fig. 3L). LiCl-treated stage 21 embryos show a complete absence of lens formation: no anterior segment thickening, lentigenic cells, or lens tissue. These data suggest that ectopic Wnt pathway activation inhibits lens and anterior segment development (Fig. 3L’, Supplemental Figure 5A). CHIR99021 treatment showed similar phenotypes (Supplemental Figure 5A). We assessed LiCl treated and control animals for cell death and find little difference between control and treated eyes suggesting that toxicity is unlikely the reason for these phenotypic changes (Supplemental Figure 5B). Wnt antagonist treatments (Quercetin) starting at stage 21 show lens development unaffected (Supplemental Figure 5C).

We were interested in the consequence of activating or inhibiting the Wnt pathway on lens development after the beginning of lentigenic cell differentiation. We performed the same 24-h LiCl exposure at stage 23 and find the lens smaller and the anterior segment less thick than control animals, but lentigenic cells and lens tissue remain identifiable. This suggests that ectopic Wnt signaling does not impact cell identity in differentiated lentigenic cells (Fig. 3M, M’). In Quercetin-treated animals starting at stage 23, the anterior segment shows minor organizational defects, but lens development appears unaffected (Supplemental Figure 5C).

The lack of lens growth in stage 21 treated animals may be a result of an imposed delay in lens formation or it may be a result of the loss of lens potential. To differentiate between these possibilities we allowed treated animals to recover. We bathed experimental and control embryos, at both stages 21 and 23, for 24 h, washed out the solution, and allowed animals to develop for an additional 48 h. LiCl-treated stage 21 embryos never recover a lens (Fig. 3N, 3N’) while LiCl treated stage 23 embryos do form a small but morphologically abnormal lens (Fig. 3O, O’). This abnormal lens is larger than the lens found in animals immediately fixed after treatment, suggesting that existing lentigenic cells at stage 23 continue to contribute to lens formation and growth. However, because the stage 23 treated lens is markedly smaller than the control, it suggests that further lentigenic cell differentiation is lost in treated animals. These data suggest that ectopic Wnt signaling leads to the disruption of lens potential and the lack of proper lentigenic cell differentiation.

**Fig. 4:**
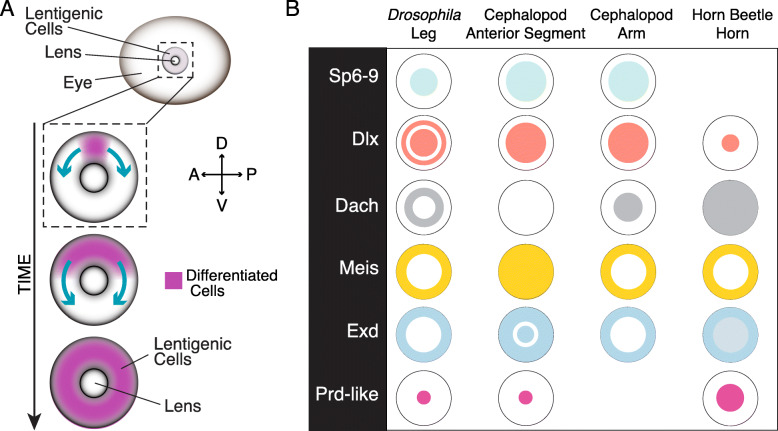
Ectopic Wnt signaling activation leads to loss of the lens. **A** Model for lentigenic cell differentiation at stage 21. LC2 lentigenic cells differentiate on the dorsal side of the eye first, with a wave moving ventrally. Type I *DpS-Crystallin* embryos have been interrupted in progress. **B** En face summary of sample radial expression of the limb patterning program across developmental contexts [14, 16, 24]

Despite the remarkable loss of the lens as a consequence of ectopic Wnt signaling, these data do not clearly distinguish between the loss of lentigenic cell fate or proper cell function, such as the growth of the cellular processes that form the lens. To assess if lentigenic cell fate is lost, we performed in situ hybridization experiments for *DpS-Crystallin* on LiCl-treated animals. We saw two types of expression phenotypes, either a significant decrease (type I) or a complete loss (type II) in *DpS-Crystallin* expression as compared to control (Fig. 4P, P’, and P”, Supplemental Figure 6). We find all *DpS-Crystallin* expression exclusively dorsal to the site of lens formation suggesting that these cells may differentiate first. These data show that ectopic Wnt signaling results in the loss of lentigenic cell fate and that our treatment may have interrupted a dorsal-to-ventral wave of differentiation in some embryos (Fig. 4A). In addition, we assessed other anterior segment markers, including *DpSix3/6* and *DpLhx1/5*, and these genes show a consistent loss of expression in the most severe phenotypes, (Supplemental Figure 6A-6C).

### Limb patterning program regulatory evolution

To address if Wnt signaling is upstream of the limb patterning program, we performed in situ hybridization of limb transcription factors after LiCl treatment (Fig. 3Q–S, Supplementary Figure 6A-6C). Similar to *DpS-Crystallin* expression, we again see a mild reduction (Type I) or loss and severe reduction (Type II) in region of expression. Our milder phenotypes, again, show a dorsal asymmetry, which can be most easily seen in *DpSP6-9A*, *DpDlx*, and *DpHbn* (Fig. 3Q, Q’, Q”; R, R’, R”; and S, S’, S”). Changes are also visible but less obvious in *DpPbx* and *DpMeis* expression, with *DpPbx* only showing a mild phenotype (Supplemental Figure 6A-6C). These data are consistent with the placement of Wnt signaling upstream of the limb patterning program in a negative regulatory role.

## Conclusion

Our findings indicate that the limb patterning program has been co-opted for the anterior segment and lens development in cephalopods and that this co-option does not have a homologous upstream regulatory relationship with Wnt signaling as found in the limb [24, 53]. This change in signaling and the known duplication of SP6-9 and the novel expression of the SP6-9a paralog in the anterior segment suggests that this duplication may be a mediator of limb patterning program co-option in the anterior segment. In vertebrates, although the limb patterning transcription factors are expressed during central nervous system development, including in the vertebrate retina, they do not have a role in lens development. Our gene expression data also suggest a role for the limb patterning program in the cephalopod nervous system, including the retina. It is known that *SP6-9* and *Dlx* are required for proper regeneration of the lens-less eye in the Planarian *Schmidtea mediterranea*, supporting an ancestral role in the Lophotrochozoa for these genes in eye formation [18]. The co-option of this network in the cephalopod lens may suggest an elaboration of the ancestral nervous system or retinal tissue [58]. This is also supported by lineage tracing data, where, early in squid development, anterior segment tissue is derived from epithelial cells contiguous with the neighboring retinal primordia [46]. In the vertebrate case, cranial ectodermal placodes are the developmental origin of the lens [59]. The vertebrate retina is derived from evagination of forebrain neurectoderm making it unlikely that the lens evolved as an elaboration of retinal tissue [60]. Together this suggests that the convergent evolution of complex phenotypes relies on a diversity of developmental origins.

Finally, with little similarity between limb and lens, our work suggests that the function of the limb patterning program in a limbless ancestor may have been a more generic developmental function than outgrowth. Considering present findings, previous work, and hypotheses, we conclude that the ability to pattern in a radial fashion, as previously proposed, is a more inclusive and likely ancestral function (Fig. 4B) [2, 30]. This work shows the cephalopod lens to be a unique context for future investigation of comparative regulatory changes responsible for co-option, and for identifying the regulatory mechanisms responsible for the emergent radial pattern found in embryos across species.

## Methods

### Animal Husbandry

*Doryteuthis pealeii* egg sacks were obtained from the Marine Biological Labs during the summer breading season. Egg sacks were kept in well-aerated 20-gallon aquarium tanks of artificial seawater at 32–35 ppt at a pH of 8 at 20 °C. To maintain proper aeration in tanks, some embryo egg masses were kept in mesh baskets accompanied with an aeration stone. Although not required, European guidelines for cephalopod research were followed.

### Histology and TUNEL staining

Embryos were fixed at 4 °C overnight in 4% paraformaldehyde (PFA) in filter-sterilized seawater. After fixation embryos were transitioned into 15% and 30% sucrose and embedded in Tissue Freezing Medium and stored at − 80 °C. Embryos were cryosectioned in 12-μm sections, stained with Sytox-Green 1:1000 and Phalloidin 555 1:300 in PBS overnight (Molecular Probes). TUNEL stained tissue was processed after sectioning using the Click-iT TUNEL Alexa Fluor 488 kit according to the manufacturer’s instructions (Invitrogen). Embryos were mounted in VECTASHIELD Hardset antifade mounting medium and imaged on a Zeiss 880 confocal.

### Homolog Identification and Phylogenetics

Genes were preliminarily identified using reciprocal BLAST with *Mus musculus* and *Drosophila melanogaster* sequences as bait with the exception of S-Crystallin where previous *Doryteuthis opalescens* sequences were also used [61]. Top hits in the *D. pealeii* transcriptome were trimmed for coding sequence and translated to amino acid sequences. To find related sequences, BLASTp was used, searching only the RefSeq protein database in NCBI filtered for vertebrate and arthropod models, as well as spiralian models when published annotated sequences could be found. The top hits of each gene name were downloaded and aligned with our *D. pealeii* sequences for each tree using MAFFT in Geneious [62]. To check sequence redundancy and proper outgroups quick trees were made using FastTree. We constructed maximum-likelihood trees on the FASRC Cannon cluster supported by the FAS Division of Science Research Computing Group at Harvard University [63]. Using PTHREADS RAxML v.8.2.10, we ran the option for rapid bootstrapping with search for best maximum likelihood tree, resampling with 1000 bootstrap replicates, the PROTGAMMAAUTO model of amino acid substitution, and otherwise default parameters [64]. Fasta alignments, Nexus tree files are found at doi:10.5061/dryad.vhhmgqnvf. All PDF versions of the trees are found in Supplemental Figure 1.

### Cloning and probe synthesis

Embryos stage 21–29 were crushed in TRIzol reagent. RNA was extracted using standard phenol-chloroform extraction with a clean-up using the Qiagen RNeasy Micro kit. cDNA was synthesized using iScript (Bio-Rad) according to manufacturer protocols. Primers were designed using Primer3 in the Geneious software package from available transcriptomic data (Koenig et al., 2016). PCR products were ligated into the Pgem-T Easy plasmid and isolated using the Qiagen miniprep kit. Plasmids were linearized using restriction enzymes. Sense and anti-sense probes were synthesized using T7 and SP6 polymerase with digoxygenin-labeled nucleotides.

### In situ hybridization

Embryos were fixed as previously described [46] and were dehydrated in 100% ethanol and stored at − 20 °C. Whole-mount in situ hybridization was performed as previously described [46]. Embryos were imaged using a Zeiss Axio Zoom.V16. Embryos were fixed for sectioning overnight in 4% PFA in artificial seawater and dehydrated in 100% ethanol. Embryos were transitioned into histoclear and embedded in paraffin. Embryos were sectioned on a Leica RM2235 microtome in 5-μm sections. Sections were dewaxed for in situ in Histoclear, rehydrated through an EtOH series, and re-fixed for 5 min at 4 °C in 4% PFA in PBS. Embryos were exposed to Proteinase K for 20 min at 37 °C and then quenched with glycine. The embryos were then de-acetylated with acetic anhydride. Slides were then pre-hybridized at 65 °C for 30–60 min and then exposed to probe overnight. Slides were washed in 50% formamide/1× SSC/0.1% Tween-20 hybridization buffer twice, then twice in 1× SSC, .2× SSC, and 0.02× SSC, all at 70 °C. The slides were then washed at room temperature in MABT three times and blocked in Roche Blocking Buffer for an hour. Slides were incubated in Anti-Dig antibody (Roche) at 1/4000 overnight at 4 °C. Slides were washed with MABT and then placed in AP reaction buffer. Slides were then exposed to BCIP/NBT solution until reacted and stopped in PBS. Slides were counterstained with Sytox-Green 1:1000 overnight. Slides mounted in ImmunoHistoMount (Abcam) and imaged on a Zeiss Axioscope. *DpS-Crystallin* embryo in situs were transitioned to sucrose and embedded after imaging in whole-mount. Embryos were image on a Zeiss Axioscope.

### Ex ovo experimental culture

Ex ovo culture was performed as previously described [46]. Embryos were bathed in .25 M, .15 M, and .07 M LiCl; 100 nm, 250 nm, and 500 nm concentration of Wnt Agonist (CHIR99021); and 25 μM, 50 μM, and 100 μM Quercetin in Pen-Step filter-sterilized seawater to determine a working concentration. Control animals were bathed in equivalent amounts of DMSO or Pen-Strep alone.

## Supplementary Information

**Additional file 1.** includes supplemental Figures 1-6. Supplemental Figure 1 is Maximum-likelihood phylogenetic trees for genes identified in this study. Supplemental Figure 2 is limb network supplemental data. Supplemental Figure 3 is targeted image enlargement of anterior segment gene expression. Supplemental Figure 4 is Wnt signaling expression supplemental data. Supplemental Figure 5 is Wnt agonist and antagonist supplemental data. Supplemental Figure 6 Supplemental in situ hybridization data and quantification. Supplemental Table 1 is are the primer sequences used to clone the genes included in this study.

## Data Availability

All sequences generated and analyzed in this study have been deposited in NCBI’s GenBank database under accession numbers MZ020516-MZ020549. All multiple sequence alignments and phylogenetic trees are available at doi:10.5061/dryad.vhhmgqnvf [65].

## Abbreviations

*a*: Arms
*aco*: Anterior chamber organ
*as*: Anterior segment
*cc*: Cup cells
*dm*: Distal-marginal cells
*e*: Eye
*f*: Funnel
*l*: Lens
*lc2*: LC2 lentigenic cell population
*lp*: Lip
*m*: Mantle
*mo*: Mouth
*pm*: Proximal-marginal cells
*rp*: Retina placode
r: Retina
*y*: Yolk
