# Supplementary material for: Co-option of the limb patterning program in cephalopod eye development
Source: BMC Biol. 2022 Jan 5;20:1. doi: 10.1186/s12915-021-01182-2 (PMC8728989; doi:10.1186/s12915-021-01182-2)
Supplement: Supplementary file 1 — Additional file 1. includes supplemental Figures 1-6. Supplemental Figure 1 is Maximum-likelihood phylogenetic trees for genes identified in this study. Supplemental Figure 2 is limb network supplemental data. Supplemental Figure 3 is targeted image enlargement of anterior segment gene expression. Supplemental Figure 4 is Wnt signaling expression supplemental data. Supplemental Figure 5 is Wnt agonist and antagonist supplemental data. Supplemental Figure 6 Supplemental in situ hybridization data and quantification. Supplemental Table 1 is are the primer sequences used to clone the genes included in this study. [file 12915_2021_1182_MOESM1_ESM.pdf]

Supplementary Information

Fig. S1. Maximum-likelihood phylogenetic trees for genes identified in this study

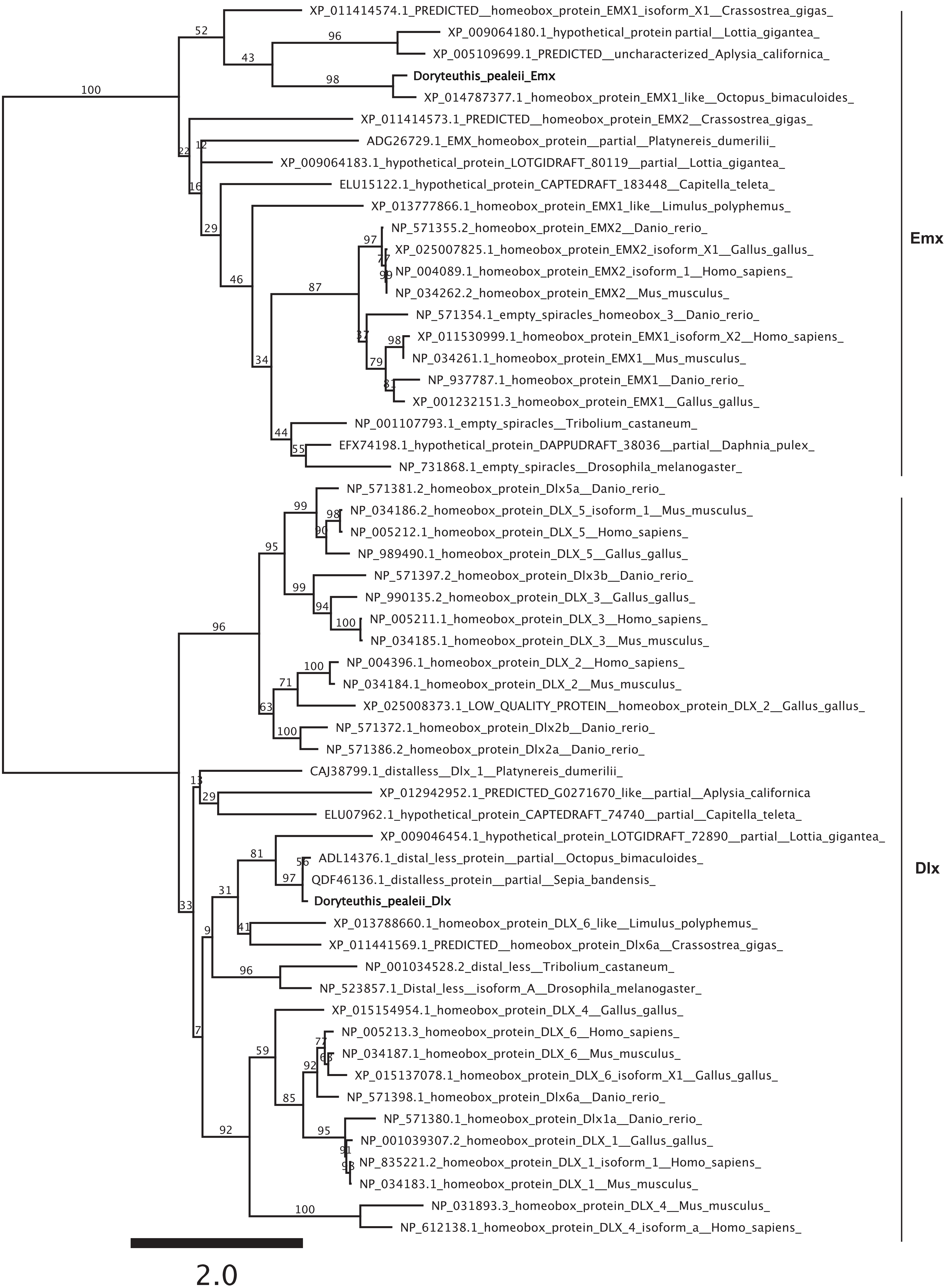

XP_011414574.1_PREDICTED__homeobox_protein_EMX1_isoform_X1__Crassostrea_gigas_
XP_009064180.1_hypothetical_protein partial__Lottia_gigantea_
XP_005109699.1_PREDICTED__uncharacterized_Aplysia_californica_
Doryteuthis_pealeii_Emx
XP_014787377.1_homeobox_protein_EMX1_like__Octopus_bimaculoides_
XP_011414573.1_PREDICTED__homeobox_protein_EMX2__Crassostrea_gigas_
ADG26729.1_EMX_homeobox_protein__partial__Platynereis_dumerilii_
XP_009064183.1_hypothetical_protein_LOTGIDRAFT_80119__partial__Lottia_gigantea_
ELU15122.1_hypothetical_protein_CAPTEDRAFT_183448__Capitella_teleta_
XP_013777866.1_homeobox_protein_EMX1_like__Limulus_polyphemus_
NP_571355.2_homeobox_protein_EMX2__Danio_rerio_
XP_025007825.1_homeobox_protein_EMX2_isoform_X1__Gallus_gallus_
NP_004089.1_homeobox_protein_EMX2_isoform_1__Homo_sapiens_
NP_034262.2_homeobox_protein_EMX2__Mus_musculus_
NP_571354.1_empty_spiracles_homeobox_3__Danio_rerio_
XP_011530999.1_homeobox_protein_EMX1_isoform_X2__Homo_sapiens_
NP_034261.1_homeobox_protein_EMX1__Mus_musculus_
NP_937787.1_homeobox_protein_EMX1__Danio_rerio_
XP_001232151.3_homeobox_protein_EMX1__Gallus_gallus_
NP_001107793.1_empty_spiracles__Tribolium_castaneum_
EFX74198.1_hypothetical_protein_DAPPUDRAFT_38036__partial__Daphnia_pulex_
NP_731868.1_empty_spiracles__Drosophila_melanogaster_
NP_571381.2_homeobox_protein_Dlx5a__Danio_rerio_
NP_034186.2_homeobox_protein_DLX_5_isoform_1__Mus_musculus_
NP_005212.1_homeobox_protein_DLX_5__Homo_sapiens_
NP_989490.1_homeobox_protein_DLX_5__Gallus_gallus_
NP_571397.2_homeobox_protein_Dlx3b__Danio_rerio_
NP_990135.2_homeobox_protein_DLX_3__Gallus_gallus_
NP_005211.1_homeobox_protein_DLX_3__Homo_sapiens_
NP_034185.1_homeobox_protein_DLX_3__Mus_musculus_
NP_004396.1_homeobox_protein_DLX_2__Homo_sapiens_
NP_034184.1_homeobox_protein_DLX_2__Mus_musculus_
XP_025008373.1_LOW_QUALITY_PROTEIN__homeobox_protein_DLX_2__Gallus_gallus_
NP_571372.1_homeobox_protein_Dlx2b__Danio_rerio_
NP_571386.2_homeobox_protein_Dlx2a__Danio_rerio_
CAJ38799.1_distalless__Dlx_1__Platynereis_dumerilii_
XP_012942952.1_PREDICTED_G0271670_like__partial__Aplysia_californica
ELU07962.1_hypothetical_protein_CAPTEDRAFT_74740__partial__Capitella_teleta_
XP_009046454.1_hypothetical_protein_LOTGIDRAFT_72890__partial__Lottia_gigantea_
ADL14376.1_distal_less_protein__partial__Octopus_bimaculoides_
QDF46136.1_distalless_protein__partial__Sepia_bandensis_
Doryteuthis_pealeii_Dlx
XP_013788660.1_homeobox_protein_DLX_6_like__Limulus_polyphemus_
XP_011441569.1_PREDICTED__homeobox_protein_Dlx6a__Crassostrea_gigas_
NP_001034528.2_distal_less__Tribolium_castaneum_
NP_523857.1_Distal_less__isoform_A__Drosophila_melanogaster_
XP_015154954.1_homeobox_protein_DLX_4__Gallus_gallus_
NP_005213.3_homeobox_protein_DLX_6__Homo_sapiens_
NP_034187.1_homeobox_protein_DLX_6__Mus_musculus_
XP_015137078.1_homeobox_protein_DLX_6_isoform_X1__Gallus_gallus_
NP_571398.1_homeobox_protein_Dlx6a__Danio_rerio_
NP_571380.1_homeobox_protein_Dlx1a__Danio_rerio_
NP_001039307.2_homeobox_protein_DLX_1__Gallus_gallus_
NP_835221.2_homeobox_protein_DLX_1_isoform_1__Homo_sapiens_
NP_034183.1_homeobox_protein_DLX_1__Mus_musculus_
NP_031893.3_homeobox_protein_DLX_4__Mus_musculus_
NP_612138.1_homeobox_protein_DLX_4_isoform_a__Homo_sapiens_
Emx
Dlx
2.0

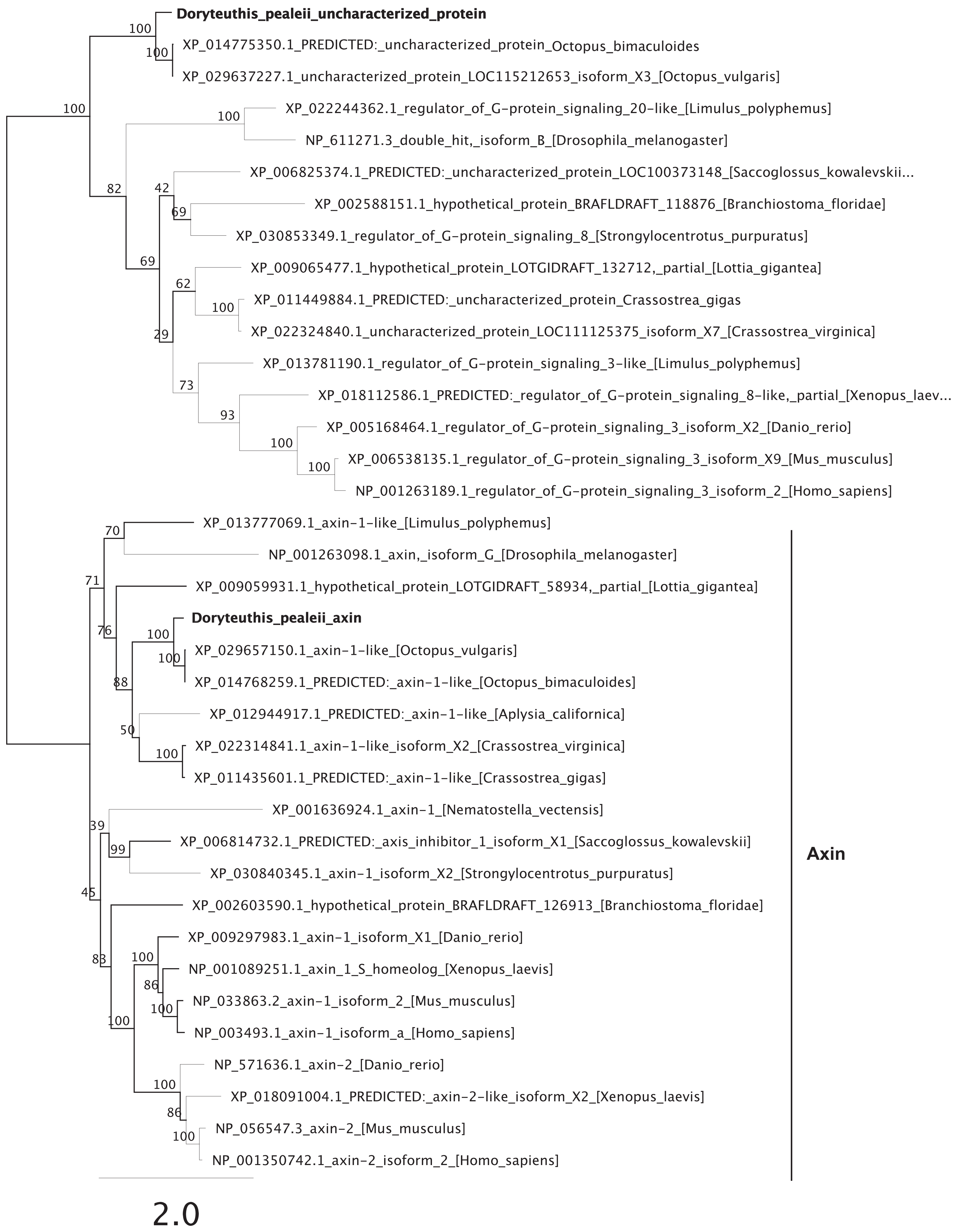

Doryteuthis_pealeii_uncharacterized_protein
XP_014775350.1_PREDICTED:_uncharacterized_protein_Octopus_bimaculoides
XP_029637227.1_uncharacterized_protein_LOC115212653_isoform_X3_[Octopus_vulgaris]
XP_022244362.1_regulator_of_G-protein_signaling_20-like_[Limulus_polyphemus]
NP_611271.3_double_hit,_isoform_B_[Drosophila_melanogaster]
XP_006825374.1_PREDICTED:_uncharacterized_protein_LOC100373148_[Saccoglossus_kowalevskii...
XP_002588151.1_hypothetical_protein_BRAFLDRAFT_118876_[Branchiostoma_floridae]
XP_030853349.1_regulator_of_G-protein_signaling_8_[Strongylocentrotus_purpuratus]
XP_009065477.1_hypothetical_protein_LOTGIDRAFT_132712,_partial_[Lottia_gigantea]
XP_011449884.1_PREDICTED:_uncharacterized_protein_Crassostrea_gigas
XP_022324840.1_uncharacterized_protein_LOC111125375_isoform_X7_[Crassostrea_virginica]
XP_013781190.1_regulator_of_G-protein_signaling_3-like_[Limulus_polyphemus]
XP_018112586.1_PREDICTED:_regulator_of_G-protein_signaling_8-like,_partial_[Xenopus_laev...
XP_005168464.1_regulator_of_G-protein_signaling_3_isoform_X2_[Danio_rerio]
XP_006538135.1_regulator_of_G-protein_signaling_3_isoform_X9_[Mus_musculus]
NP_001263189.1_regulator_of_G-protein_signaling_3_isoform_2_[Homo_sapiens]
XP_013777069.1_axin-1-like_[Limulus_polyphemus]
NP_001263098.1_axin,_isoform_G_[Drosophila_melanogaster]
XP_009059931.1_hypothetical_protein_LOTGIDRAFT_58934,_partial_[Lottia_gigantea]
Doryteuthis_pealeii_axin
XP_029657150.1_axin-1-like_[Octopus_vulgaris]
XP_014768259.1_PREDICTED:_axin-1-like_[Octopus_bimaculoides]
XP_012944917.1_PREDICTED:_axin-1-like_[Aplysia_californica]
XP_022314841.1_axin-1-like_isoform_X2_[Crassostrea_virginica]
XP_011435601.1_PREDICTED:_axin-1-like_[Crassostrea_gigas]
XP_001636924.1_axin-1_[Nematostella_vectensis]
XP_006814732.1_PREDICTED:_axis_inhibitor_1_isoform_X1_[Saccoglossus_kowalevskii]
XP_030840345.1_axin-1_isoform_X2_[Strongylocentrotus_purpuratus]
XP_002603590.1_hypothetical_protein_BRAFLDRAFT_126913_[Branchiostoma_floridae]
XP_009297983.1_axin-1_isoform_X1_[Danio_rerio]
NP_001089251.1_axin_1_S_homeolog_[Xenopus_laevis]
NP_033863.2_axin-1_isoform_2_[Mus_musculus]
NP_003493.1_axin-1_isoform_a_[Homo_sapiens]
NP_571636.1_axin-2_[Danio_rerio]
XP_018091004.1_PREDICTED:_axin-2-like_isoform_X2_[Xenopus_laevis]
NP_056547.3_axin-2_[Mus_musculus]
NP_001350742.1_axin-2_isoform_2_[Homo_sapiens]
Axin
2.0

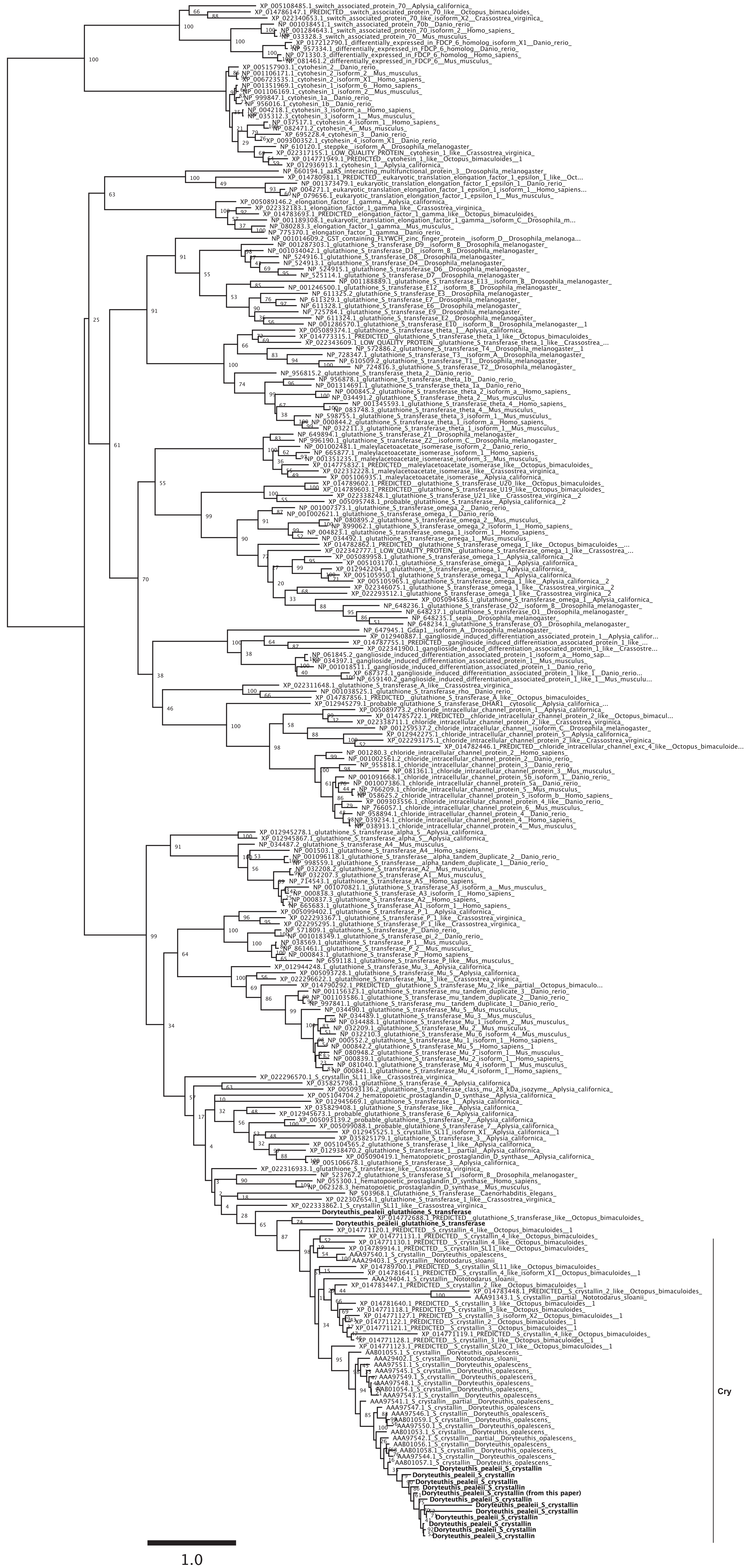
Doryteuthis_pealeii_glutathione_S_transferase
Doryteuthis_pealeii_glutathione_S_transferase
Doryteuthis_pealeii_S_crystallin
Doryteuthis_pealeii_S_crystallin (from this paper)
Cry
1.0

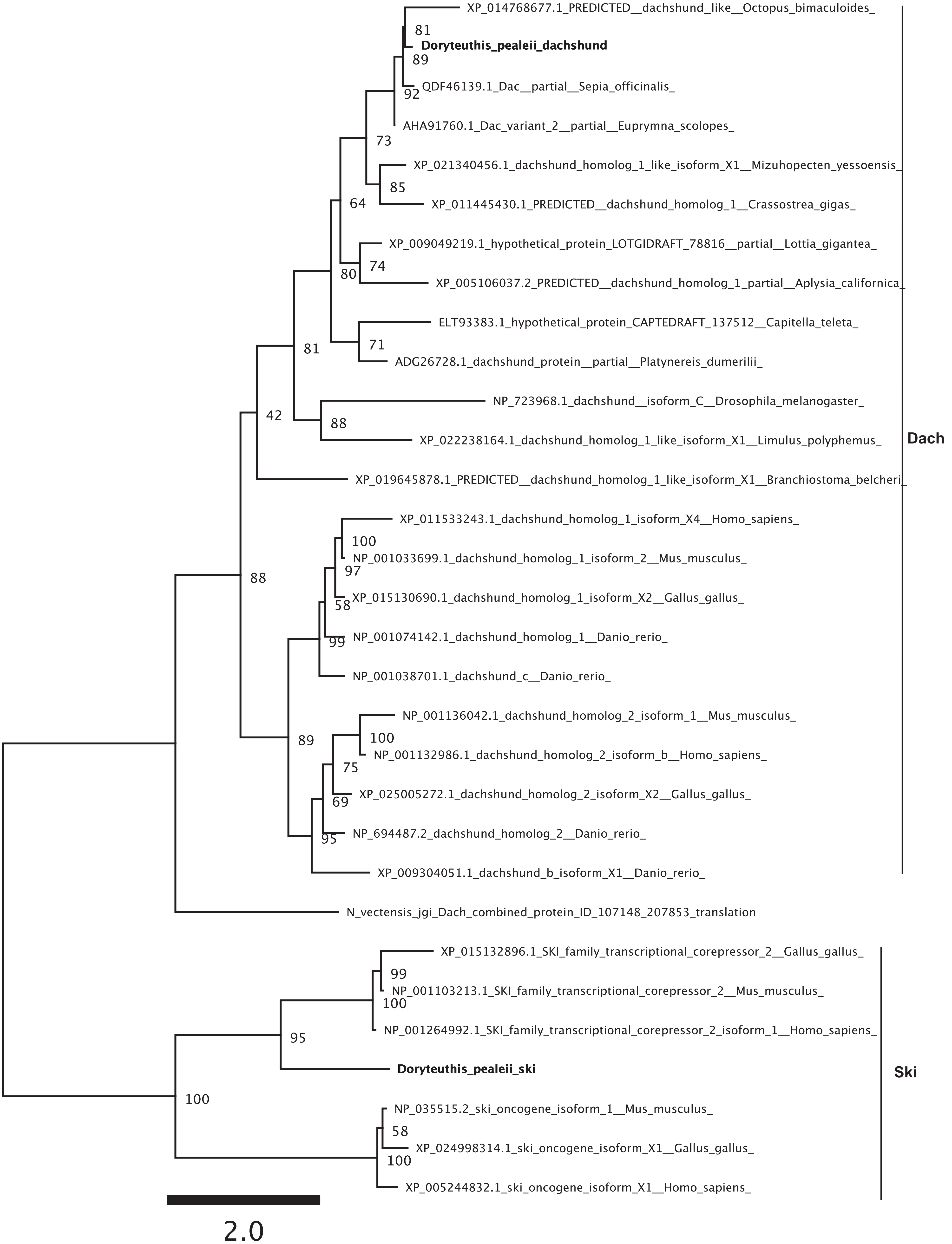

XP_014768677.1_PREDICTED__dachshund_like__Octopus_bimaculoides_
81
Doryteuthis_pealeii_dachshund
89
QDF46139.1_Dac__partial__Sepia_officinalis_
92
AHA91760.1_Dac_variant_2__partial__Euprymna_scolopes_
73
XP_021340456.1_dachshund_homolog_1_like_isoform_X1__Mizuhopecten_yessoensis_
85
64
XP_011445430.1_PREDICTED__dachshund_homolog_1__Crassostrea_gigas_
XP_009049219.1_hypothetical_protein_LOTGIDRAFT_78816__partial__Lottia_gigantea_
74
80
XP_005106037.2_PREDICTED__dachshund_homolog_1_partial__Aplysia_californica_
ELT93383.1_hypothetical_protein_CAPTEDRAFT_137512__Capitella_teleta_
71
81
ADG26728.1_dachshund_protein__partial__Platynereis_dumerilii_
NP_723968.1_dachshund__isoform_C__Drosophila_melanogaster_
42
88
XP_022238164.1_dachshund_homolog_1_like_isoform_X1__Limulus_polyphemus_
Dach
XP_019645878.1_PREDICTED__dachshund_homolog_1_like_isoform_X1__Branchiostoma_belcheri_
XP_011533243.1_dachshund_homolog_1_isoform_X4__Homo_sapiens_
100
NP_001033699.1_dachshund_homolog_1_isoform_2__Mus_musculus_
97
88
XP_015130690.1_dachshund_homolog_1_isoform_X2__Gallus_gallus_
58
NP_001074142.1_dachshund_homolog_1__Danio_rerio_
99
NP_001038701.1_dachshund_c__Danio_rerio_
NP_001136042.1_dachshund_homolog_2_isoform_1__Mus_musculus_
89
100
NP_001132986.1_dachshund_homolog_2_isoform_b__Homo_sapiens_
75
XP_025005272.1_dachshund_homolog_2_isoform_X2__Gallus_gallus_
69
NP_694487.2_dachshund_homolog_2__Danio_rerio_
95
XP_009304051.1_dachshund_b_isoform_X1__Danio_rerio_
N_vectensis_jgi_Dach_combined_protein_ID_107148_207853_translation
XP_015132896.1_SKI_family_transcriptional_corepressor_2__Gallus_gallus_
99
NP_001103213.1_SKI_family_transcriptional_corepressor_2__Mus_musculus_
100
NP_001264992.1_SKI_family_transcriptional_corepressor_2_isoform_1__Homo_sapiens_
95
Doryteuthis_pealeii_ski
Ski
100
NP_035515.2_ski_oncogene_isoform_1__Mus_musculus_
58
XP_024998314.1_ski_oncogene_isoform_X1__Gallus_gallus_
100
XP_005244832.1_ski_oncogene_isoform_X1__Homo_sapiens_
2.0

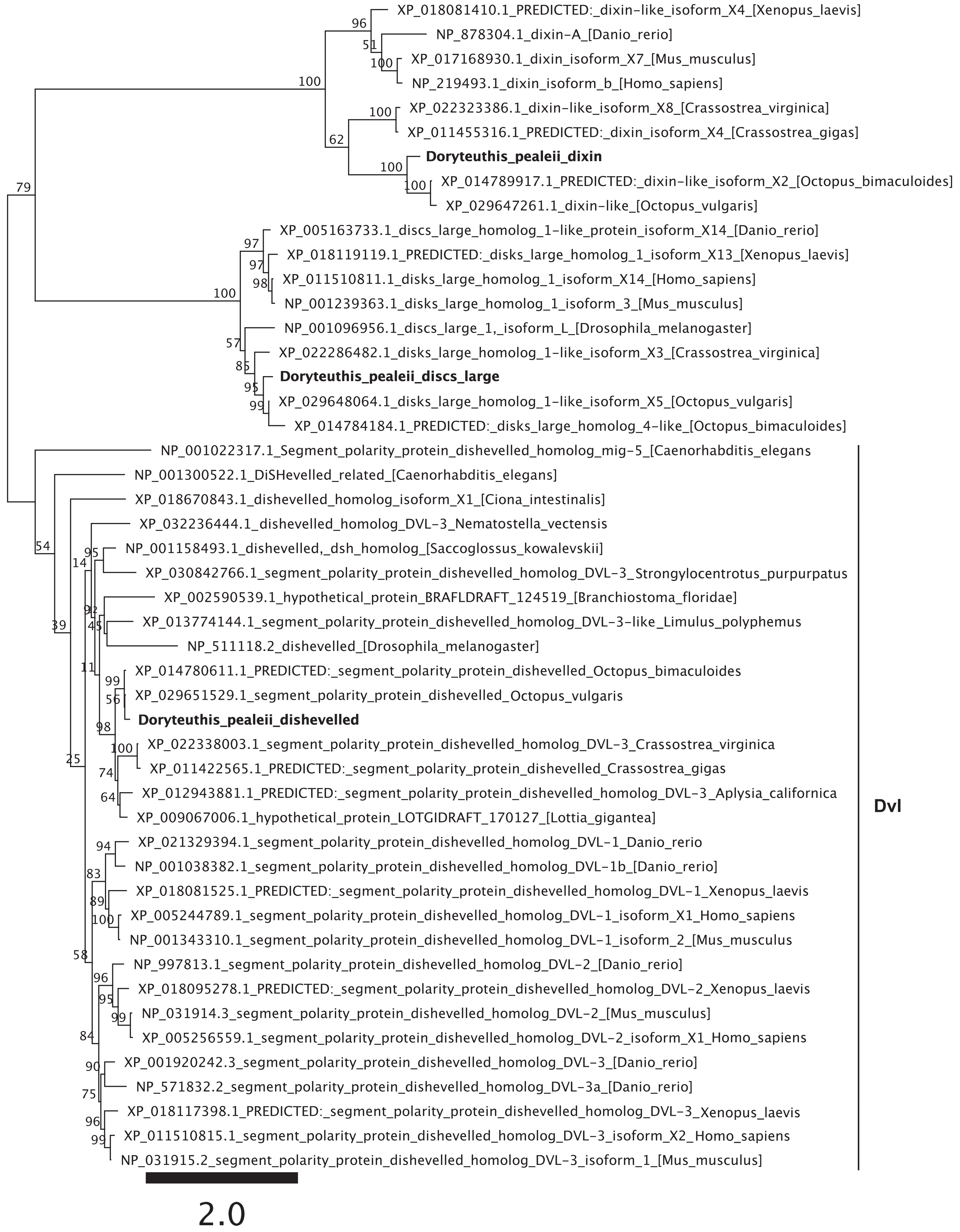

XP_018081410.1_PREDICTED:_dixin-like_isoform_X4_[Xenopus_laevis]
NP_878304.1_dixin-A_[Danio_rerio]
XP_017168930.1_dixin_isoform_X7_[Mus_musculus]
NP_219493.1_dixin_isoform_b_[Homo_sapiens]
XP_022323386.1_dixin-like_isoform_X8_[Crassostrea_virginica]
XP_011455316.1_PREDICTED:_dixin_isoform_X4_[Crassostrea_gigas]
Doryteuthis_pealeii_dixin
XP_014789917.1_PREDICTED:_dixin-like_isoform_X2_[Octopus_bimaculoides]
XP_029647261.1_dixin-like_[Octopus_vulgaris]
XP_005163733.1_discs_large_homolog_1-like_protein_isoform_X14_[Danio_rerio]
XP_018119119.1_PREDICTED:_disks_large_homolog_1_isoform_X13_[Xenopus_laevis]
XP_011510811.1_disks_large_homolog_1_isoform_X14_[Homo_sapiens]
NP_001239363.1_disks_large_homolog_1_isoform_3_[Mus_musculus]
NP_001096956.1_discs_large_1,_isoform_L_[Drosophila_melanogaster]
XP_022286482.1_disks_large_homolog_1-like_isoform_X3_[Crassostrea_virginica]
Doryteuthis_pealeii_discs_large
XP_029648064.1_disks_large_homolog_1-like_isoform_X5_[Octopus_vulgaris]
XP_014784184.1_PREDICTED:_disks_large_homolog_4-like_[Octopus_bimaculoides]
NP_001022317.1_Segment_polarity_protein_dishevelled_homolog_mig-5_[Caenorhabditis_elegans
NP_001300522.1_DiSHevelled_related_[Caenorhabditis_elegans]
XP_018670843.1_dishevelled_homolog_isoform_X1_[Ciona_intestinalis]
XP_032236444.1_dishevelled_homolog_DVL-3_Nematostella_vectensis
NP_001158493.1_dishevelled,_dsh_homolog_[Saccoglossus_kowalevskii]
XP_030842766.1_segment_polarity_protein_dishevelled_homolog_DVL-3_Strongylocentrotus_purpurpatus
XP_002590539.1_hypothetical_protein_BRAFLDRAFT_124519_[Branchiostoma_floridae]
XP_013774144.1_segment_polarity_protein_dishevelled_homolog_DVL-3-like_Limulus_polyphemus
NP_511118.2_dishevelled_[Drosophila_melanogaster]
XP_014780611.1_PREDICTED:_segment_polarity_protein_dishevelled_Octopus_bimaculoides
XP_029651529.1_segment_polarity_protein_dishevelled_Octopus_vulgaris
Doryteuthis_pealeii_dishevelled
XP_022338003.1_segment_polarity_protein_dishevelled_homolog_DVL-3_Crassostrea_virginica
XP_011422565.1_PREDICTED:_segment_polarity_protein_dishevelled_Crassostrea_gigas
XP_012943881.1_PREDICTED:_segment_polarity_protein_dishevelled_homolog_DVL-3_Aplysia_californica
XP_009067006.1_hypothetical_protein_LOTGIDRAFT_170127_[Lottia_gigantea]
XP_021329394.1_segment_polarity_protein_dishevelled_homolog_DVL-1_Danio_rerio
NP_001038382.1_segment_polarity_protein_dishevelled_homolog_DVL-1b_[Danio_rerio]
XP_018081525.1_PREDICTED:_segment_polarity_protein_dishevelled_homolog_DVL-1_Xenopus_laevis
XP_005244789.1_segment_polarity_protein_dishevelled_homolog_DVL-1_isoform_X1_Homo_sapiens
NP_001343310.1_segment_polarity_protein_dishevelled_homolog_DVL-1_isoform_2_[Mus_musculus
NP_997813.1_segment_polarity_protein_dishevelled_homolog_DVL-2_[Danio_rerio]
XP_018095278.1_PREDICTED:_segment_polarity_protein_dishevelled_homolog_DVL-2_Xenopus_laevis
NP_031914.3_segment_polarity_protein_dishevelled_homolog_DVL-2_[Mus_musculus]
XP_005256559.1_segment_polarity_protein_dishevelled_homolog_DVL-2_isoform_X1_Homo_sapiens
XP_001920242.3_segment_polarity_protein_dishevelled_homolog_DVL-3_[Danio_rerio]
NP_571832.2_segment_polarity_protein_dishevelled_homolog_DVL-3a_[Danio_rerio]
XP_018117398.1_PREDICTED:_segment_polarity_protein_dishevelled_homolog_DVL-3_Xenopus_laevis
XP_011510815.1_segment_polarity_protein_dishevelled_homolog_DVL-3_isoform_X2_Homo_sapiens
NP_031915.2_segment_polarity_protein_dishevelled_homolog_DVL-3_isoform_1_[Mus_musculus]
Dvl
2.0

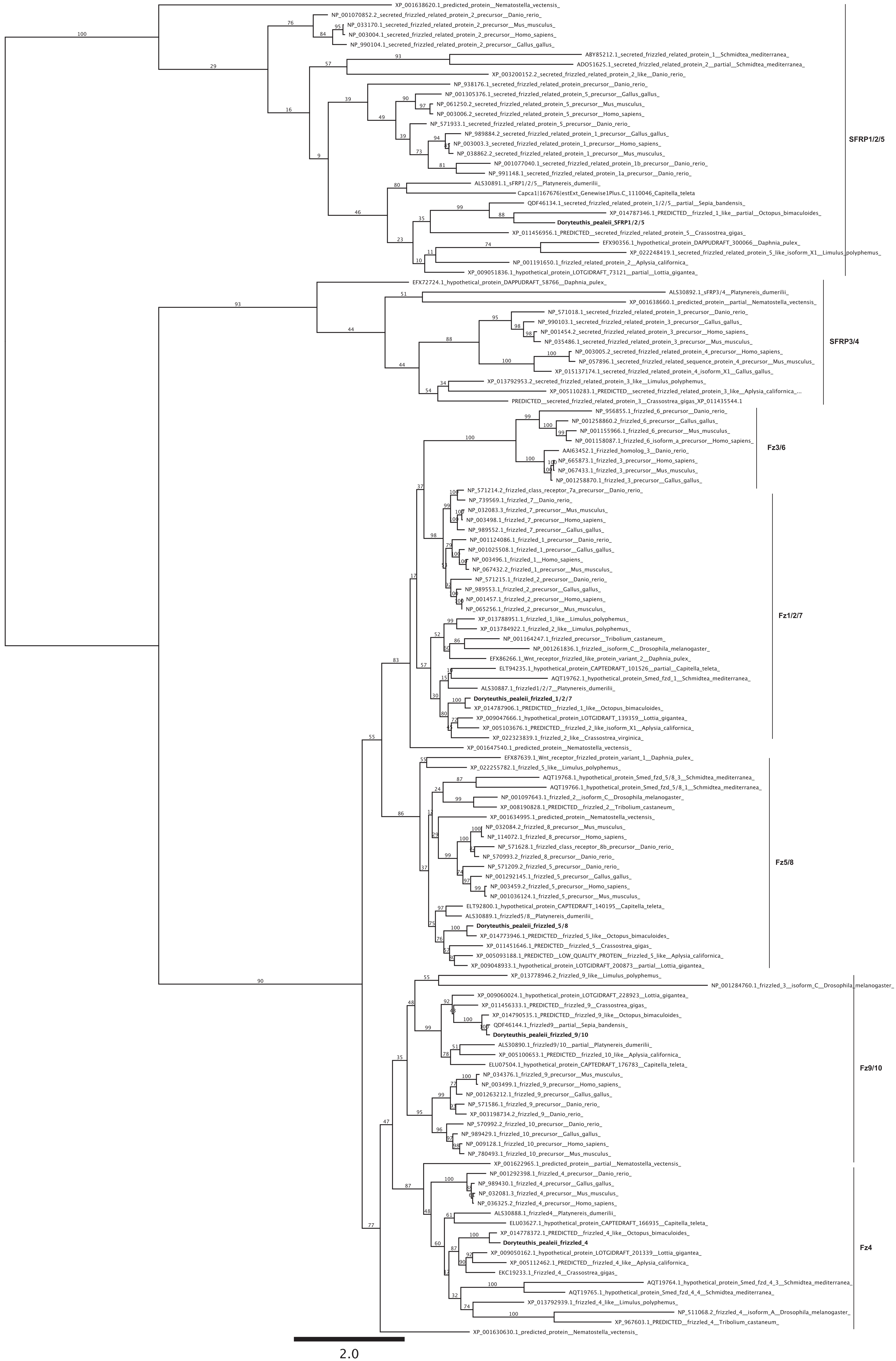
XP_001638620.1_predicted_protein__Nematostella_vectensis_
NP_001070852.2_secreted_frizzled_related_protein_2_precursor__Danio_rerio_
NP_033170.1_secreted_frizzled_related_protein_2_precursor__Mus_musculus_
NP_003004.1_secreted_frizzled_related_protein_2_precursor__Homo_sapiens_
NP_990104.1_secreted_frizzled_related_protein_2_precursor__Gallus_gallus_
ABY85212.1_secreted_frizzled_related_protein_1__Schmidtea_mediterranea_
ADO51625.1_secreted_frizzled_related_protein_2__partial__Schmidtea_mediterranea_
XP_003200152.2_secreted_frizzled_related_protein_2_like__Danio_rerio_
NP_938176.1_secreted_frizzled_related_protein_precursor__Danio_rerio_
NP_001305376.1_secreted_frizzled_related_protein_5_precursor__Gallus_gallus_
NP_061250.2_secreted_frizzled_related_protein_5_precursor__Mus_musculus_
NP_003006.2_secreted_frizzled_related_protein_5_precursor__Homo_sapiens_
NP_571933.1_secreted_frizzled_related_protein_5_precursor__Danio_rerio_
NP_989884.2_secreted_frizzled_related_protein_1_precursor__Gallus_gallus_
NP_003003.3_secreted_frizzled_related_protein_1_precursor__Homo_sapiens_
NP_038862.2_secreted_frizzled_related_protein_1_precursor__Mus_musculus_
NP_001077040.1_secreted_frizzled_related_protein_1b_precursor__Danio_rerio_
NP_991148.1_secreted_frizzled_related_protein_1a_precursor__Danio_rerio_
ALS30891.1_sFRP1/2/5__Platynereis_dumerilii_
Capca1|167676|estExt_Genewise1Plus.C_1110046_Capitella_teleta
QDF46134.1_secreted_frizzled_related_protein_1/2/5__partial__Sepia_bandensis_
XP_014787346.1_PREDICTED__frizzled_1_like__partial__Octopus_bimaculoides_
Doryteuthis_pealeii_SFRP1/2/5
XP_011456956.1_PREDICTED__secreted_frizzled_related_protein_5__Crassostrea_gigas_
EFX90356.1_hypothetical_protein_DAPPUDRAFT_300066__Daphnia_pulex_
XP_022248419.1_secreted_frizzled_related_protein_5_like_isoform_X1__Limulus_polyphemus_
NP_001191650.1_frizzled_related_protein_2__Aplysia_californica_
XP_009051836.1_hypothetical_protein_LOTGIDRAFT_73121__partial__Lottia_gigantea_
SFRP1/2/5
EFX72724.1_hypothetical_protein_DAPPUDRAFT_58766__Daphnia_pulex_
ALS30892.1_sFRP3/4__Platynereis_dumerilii_
XP_001638660.1_predicted_protein__partial__Nematostella_vectensis_
NP_571018.1_secreted_frizzled_related_protein_3_precursor__Danio_rerio_
NP_990103.1_secreted_frizzled_related_protein_3_precursor__Gallus_gallus_
NP_001454.2_secreted_frizzled_related_protein_3_precursor__Homo_sapiens_
NP_035486.1_secreted_frizzled_related_protein_3_precursor__Mus_musculus_
NP_003005.2_secreted_frizzled_related_protein_4_precursor__Homo_sapiens_
NP_057896.1_secreted_frizzled_related_sequence_protein_4_precursor__Mus_musculus_
XP_015137174.1_secreted_frizzled_related_protein_4_isoform_X1__Gallus_gallus_
XP_013792953.2_secreted_frizzled_related_protein_3_like__Limulus_polyphemus_
XP_005110283.1_PREDICTED__secreted_frizzled_related_protein_3_like__Aplysia_californica_...
PREDICTED__secreted_frizzled_related_protein_3__Crassostrea_gigas_XP_011435544.1
SFRP3/4
NP_956855.1_frizzled_6_precursor__Danio_rerio_
NP_001258860.2_frizzled_6_precursor__Gallus_gallus_
NP_001155966.1_frizzled_6_precursor__Mus_musculus_
NP_001158087.1_frizzled_6_isoform_a_precursor__Homo_sapiens_
AAI63452.1_Frizzled_homolog_3__Danio_rerio_
NP_665873.1_frizzled_3_precursor__Homo_sapiens_
NP_067433.1_frizzled_3_precursor__Mus_musculus_
NP_001258870.1_frizzled_3_precursor__Gallus_gallus_
Fz3/6
NP_571214.2_frizzled_class_receptor_7a_precursor__Danio_rerio_
NP_739569.1_frizzled_7__Danio_rerio_
NP_032083.3_frizzled_7_precursor__Mus_musculus_
NP_003498.1_frizzled_7_precursor__Homo_sapiens_
NP_989552.1_frizzled_7_precursor__Gallus_gallus_
NP_001124086.1_frizzled_1_precursor__Danio_rerio_
NP_001025508.1_frizzled_1_precursor__Gallus_gallus_
NP_003496.1_frizzled_1__Homo_sapiens_
NP_067432.2_frizzled_1_precursor__Mus_musculus_
NP_571215.1_frizzled_2_precursor__Danio_rerio_
NP_989553.1_frizzled_2_precursor__Gallus_gallus_
NP_001457.1_frizzled_2_precursor__Homo_sapiens_
NP_065256.1_frizzled_2_precursor__Mus_musculus_
XP_013788951.1_frizzled_1_like__Limulus_polyphemus_
XP_013784922.1_frizzled_2_like__Limulus_polyphemus_
NP_001164247.1_frizzled_precursor__Tribolium_castaneum_
NP_001261836.1_frizzled__isoform_C__Drosophila_melanogaster_
EFX86266.1_Wnt_receptor_frizzled_like_protein_variant_2__Daphnia_pulex_
ELT94235.1_hypothetical_protein_CAPTEDRAFT_101526__partial__Capitella_teleta_
AQT19762.1_hypothetical_protein_Smed_fzd_1__Schmidtea_mediterranea_
ALS30887.1_frizzled1/2/7__Platynereis_dumerilii_
Doryteuthis_pealeii_frizzled_1/2/7
XP_014787906.1_PREDICTED__frizzled_1_like__Octopus_bimaculoides_
XP_009047666.1_hypothetical_protein_LOTGIDRAFT_139359__Lottia_gigantea_
XP_005103676.1_PREDICTED__frizzled_2_like_isoform_X1__Aplysia_californica_
XP_022323839.1_frizzled_2_like__Crassostrea_virginica_
XP_001647540.1_predicted_protein__Nematostella_vectensis_
Fz1/2/7
EFX87639.1_Wnt_receptor_frizzled_protein_variant_1__Daphnia_pulex_
XP_022255782.1_frizzled_5_like__Limulus_polyphemus_
AQT19768.1_hypothetical_protein_Smed_fzd_5/8_3__Schmidtea_mediterranea_
AQT19766.1_hypothetical_protein_Smed_fzd_5/8_1__Schmidtea_mediterranea_
NP_001097643.1_frizzled_2__isoform_C__Drosophila_melanogaster_
XP_008190828.1_PREDICTED__frizzled_2__Tribolium_castaneum_
XP_001634995.1_predicted_protein__Nematostella_vectensis_
NP_032084.2_frizzled_8_precursor__Mus_musculus_
NP_114072.1_frizzled_8_precursor__Homo_sapiens_
NP_571628.1_frizzled_class_receptor_8b_precursor__Danio_rerio_
NP_570993.2_frizzled_8_precursor__Danio_rerio_
NP_571209.2_frizzled_5_precursor__Danio_rerio_
NP_001292145.1_frizzled_5_precursor__Gallus_gallus_
NP_003459.2_frizzled_5_precursor__Homo_sapiens_
NP_001036124.1_frizzled_5_precursor__Mus_musculus_
ELT92800.1_hypothetical_protein_CAPTEDRAFT_140195__Capitella_teleta_
ALS30889.1_frizzled5/8__Platynereis_dumerilii_
Doryteuthis_pealeii_frizzled_5/8
XP_014773946.1_PREDICTED__frizzled_5_like__Octopus_bimaculoides_
XP_011451646.1_PREDICTED__frizzled_5__Crassostrea_gigas_
XP_005093188.1_PREDICTED__LOW_QUALITY_PROTEIN__frizzled_5_like__Aplysia_californica_
XP_009048933.1_hypothetical_protein_LOTGIDRAFT_200873__partial__Lottia_gigantea_
Fz5/8
XP_013778946.2_frizzled_9_like__Limulus_polyphemus_
NP_001284760.1_frizzled_3__isoform_C__Drosophila_melanogaster_
XP_009060024.1_hypothetical_protein_LOTGIDRAFT_228923__Lottia_gigantea_
XP_011456333.1_PREDICTED__frizzled_9__Crassostrea_gigas_
XP_014790535.1_PREDICTED__frizzled_9_like__Octopus_bimaculoides_
QDF46144.1_frizzled9__partial__Sepia_bandensis_
Doryteuthis_pealeii_frizzled_9/10
ALS30890.1_frizzled9/10__partial__Platynereis_dumerilii_
XP_005100653.1_PREDICTED__frizzled_10_like__Aplysia_californica_
ELU07504.1_hypothetical_protein_CAPTEDRAFT_176783__Capitella_teleta_
NP_034376.1_frizzled_9_precursor__Mus_musculus_
NP_003499.1_frizzled_9_precursor__Homo_sapiens_
NP_001263212.1_frizzled_9_precursor__Gallus_gallus_
NP_571586.1_frizzled_9_precursor__Danio_rerio_
XP_003198734.2_frizzled_9__Danio_rerio_
NP_570992.2_frizzled_10_precursor__Danio_rerio_
NP_989429.1_frizzled_10_precursor__Gallus_gallus_
NP_009128.1_frizzled_10_precursor__Homo_sapiens_
NP_780493.1_frizzled_10_precursor__Mus_musculus_
Fz9/10
XP_001622965.1_predicted_protein__partial__Nematostella_vectensis_
NP_001292398.1_frizzled_4_precursor__Danio_rerio_
NP_989430.1_frizzled_4_precursor__Gallus_gallus_
NP_032081.3_frizzled_4_precursor__Mus_musculus_
NP_036325.2_frizzled_4_precursor__Homo_sapiens_
ALS30888.1_frizzled4__Platynereis_dumerilii_
ELU03627.1_hypothetical_protein_CAPTEDRAFT_166935__Capitella_teleta_
XP_014778372.1_PREDICTED__frizzled_4_like__Octopus_bimaculoides_
Doryteuthis_pealeii_frizzled_4
XP_009050162.1_hypothetical_protein_LOTGIDRAFT_201339__Lottia_gigantea_
XP_005112462.1_PREDICTED__frizzled_4_like__Aplysia_californica_
EKC19233.1_Frizzled_4__Crassostrea_gigas_
AQT19764.1_hypothetical_protein_Smed_fzd_4_3__Schmidtea_mediterranea_
AQT19765.1_hypothetical_protein_Smed_fzd_4_4__Schmidtea_mediterranea_
XP_013792939.1_frizzled_4_like__Limulus_polyphemus_
NP_511068.2_frizzled_4__isoform_A__Drosophila_melanogaster_
XP_967603.1_PREDICTED__frizzled_4__Tribolium_castaneum_
Fz4
XP_001630630.1_predicted_protein__Nematostella_vectensis_
2.0

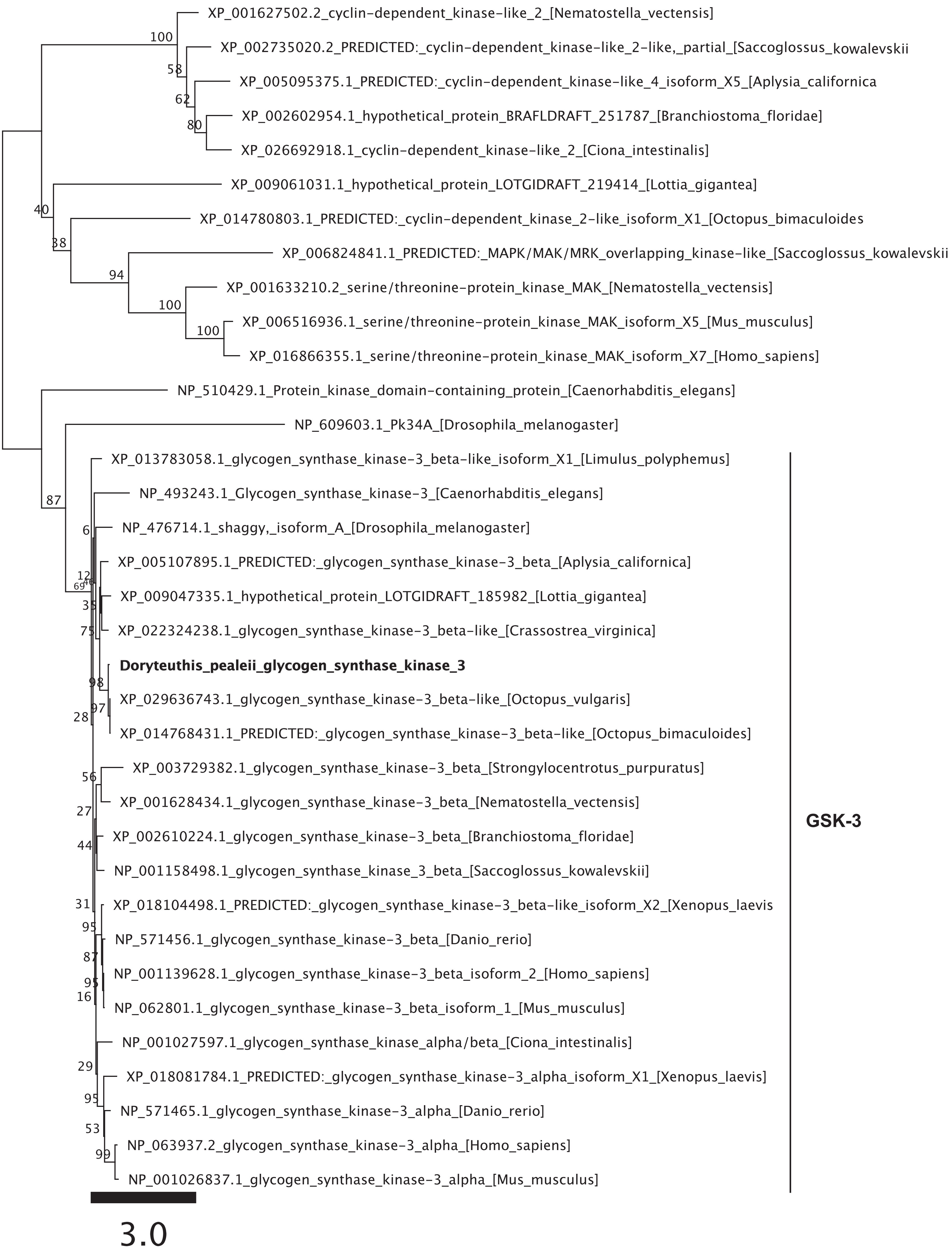

XP_001627502.2_cyclin-dependent_kinase-like_2_[Nematostella_vectensis]
XP_002735020.2_PREDICTED:_cyclin-dependent_kinase-like_2-like,_partial_[Saccoglossus_kowalevskii
XP_005095375.1_PREDICTED:_cyclin-dependent_kinase-like_4_isoform_X5_[Aplysia_californica
XP_002602954.1_hypothetical_protein_BRAFLDRAFT_251787_[Branchiostoma_floridae]
XP_026692918.1_cyclin-dependent_kinase-like_2_[Ciona_intestinalis]
XP_009061031.1_hypothetical_protein_LOTGIDRAFT_219414_[Lottia_gigantea]
XP_014780803.1_PREDICTED:_cyclin-dependent_kinase_2-like_isoform_X1_[Octopus_bimaculoides
XP_006824841.1_PREDICTED:_MAPK/MAK/MRK_overlapping_kinase-like_[Saccoglossus_kowalevskii
XP_001633210.2_serine/threonine-protein_kinase_MAK_[Nematostella_vectensis]
XP_006516936.1_serine/threonine-protein_kinase_MAK_isoform_X5_[Mus_musculus]
XP_016866355.1_serine/threonine-protein_kinase_MAK_isoform_X7_[Homo_sapiens]
NP_510429.1_Protein_kinase_domain-containing_protein_[Caenorhabditis_elegans]
NP_609603.1_Pk34A_[Drosophila_melanogaster]
XP_013783058.1_glycogen_synthase_kinase-3_beta-like_isoform_X1_[Limulus_polyphemus]
NP_493243.1_Glycogen_synthase_kinase-3_[Caenorhabditis_elegans]
NP_476714.1_shaggy,_isoform_A_[Drosophila_melanogaster]
XP_005107895.1_PREDICTED:_glycogen_synthase_kinase-3_beta_[Aplysia_californica]
XP_009047335.1_hypothetical_protein_LOTGIDRAFT_185982_[Lottia_gigantea]
XP_022324238.1_glycogen_synthase_kinase-3_beta-like_[Crassostrea_virginica]
Doryteuthis_pealeii_glycogen_synthase_kinase_3
XP_029636743.1_glycogen_synthase_kinase-3_beta-like_[Octopus_vulgaris]
XP_014768431.1_PREDICTED:_glycogen_synthase_kinase-3_beta-like_[Octopus_bimaculoides]
XP_003729382.1_glycogen_synthase_kinase-3_beta_[Strongylocentrotus_purpuratus]
XP_001628434.1_glycogen_synthase_kinase-3_beta_[Nematostella_vectensis]
XP_002610224.1_glycogen_synthase_kinase-3_beta_[Branchiostoma_floridae]
NP_001158498.1_glycogen_synthase_kinase_3_beta_[Saccoglossus_kowalevskii]
XP_018104498.1_PREDICTED:_glycogen_synthase_kinase-3_beta-like_isoform_X2_[Xenopus_laevis
NP_571456.1_glycogen_synthase_kinase-3_beta_[Danio_rerio]
NP_001139628.1_glycogen_synthase_kinase-3_beta_isoform_2_[Homo_sapiens]
NP_062801.1_glycogen_synthase_kinase-3_beta_isoform_1_[Mus_musculus]
NP_001027597.1_glycogen_synthase_kinase_alpha/beta_[Ciona_intestinalis]
XP_018081784.1_PREDICTED:_glycogen_synthase_kinase-3_alpha_isoform_X1_[Xenopus_laevis]
NP_571465.1_glycogen_synthase_kinase-3_alpha_[Danio_rerio]
NP_063937.2_glycogen_synthase_kinase-3_alpha_[Homo_sapiens]
NP_001026837.1_glycogen_synthase_kinase-3_alpha_[Mus_musculus]
GSK-3
3.0

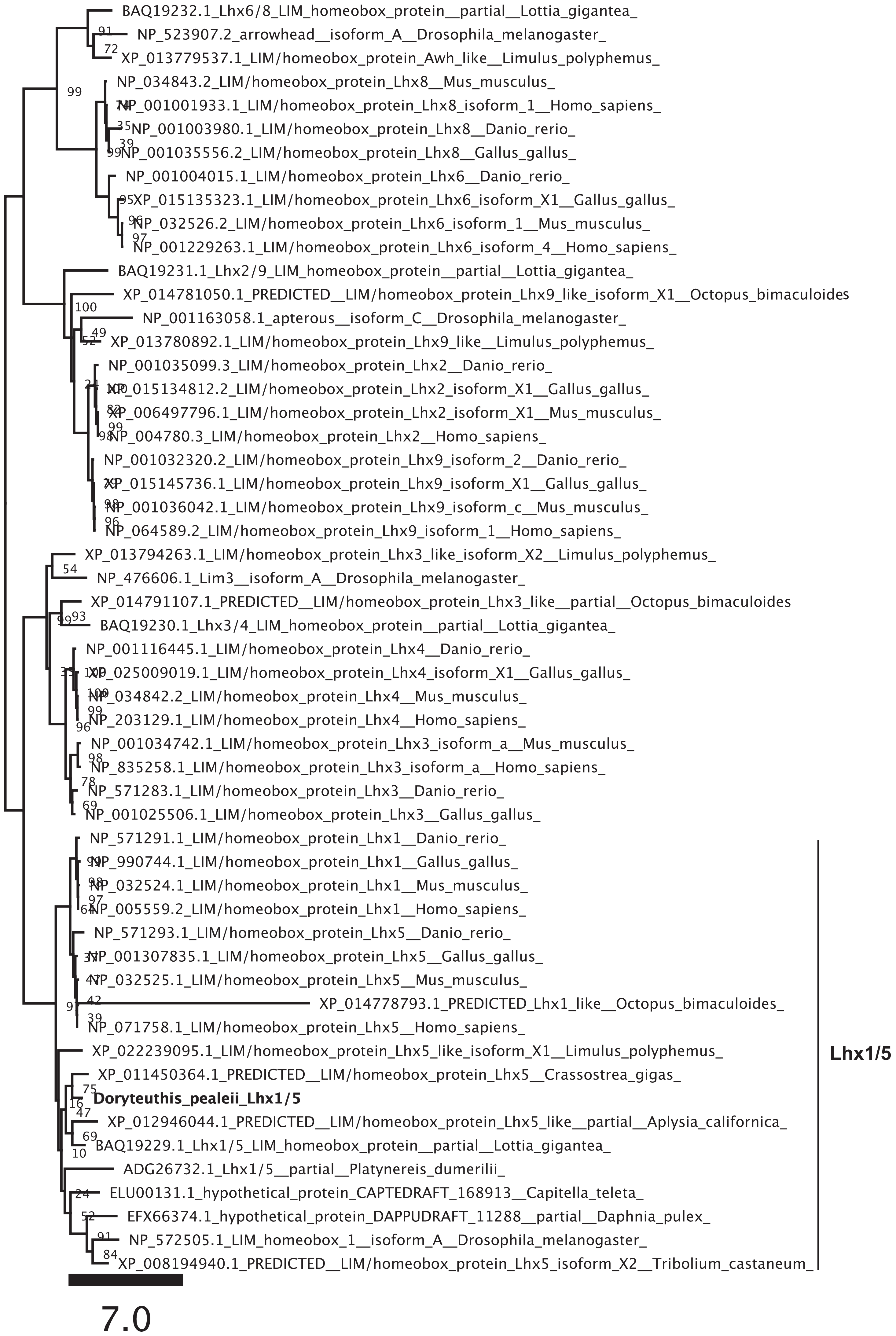
BAQ19232.1_Lhx6/8_LIM_homeobox_protein__partial__Lottia_gigantea_
NP_523907.2_arrowhead__isoform_A__Drosophila_melanogaster_
XP_013779537.1_LIM/homeobox_protein_Awh_like__Limulus_polyphemus_
NP_034843.2_LIM/homeobox_protein_Lhx8__Mus_musculus_
NP_001001933.1_LIM/homeobox_protein_Lhx8_isoform_1__Homo_sapiens_
NP_001003980.1_LIM/homeobox_protein_Lhx8__Danio_rerio_
NP_001035556.2_LIM/homeobox_protein_Lhx8__Gallus_gallus_
NP_001004015.1_LIM/homeobox_protein_Lhx6__Danio_rerio_
XP_015135323.1_LIM/homeobox_protein_Lhx6_isoform_X1__Gallus_gallus_
NP_032526.2_LIM/homeobox_protein_Lhx6_isoform_1__Mus_musculus_
NP_001229263.1_LIM/homeobox_protein_Lhx6_isoform_4__Homo_sapiens_
BAQ19231.1_Lhx2/9_LIM_homeobox_protein__partial__Lottia_gigantea_
XP_014781050.1_PREDICTED__LIM/homeobox_protein_Lhx9_like_isoform_X1__Octopus_bimaculoides
NP_001163058.1_apterous__isoform_C__Drosophila_melanogaster_
XP_013780892.1_LIM/homeobox_protein_Lhx9_like__Limulus_polyphemus_
NP_001035099.3_LIM/homeobox_protein_Lhx2__Danio_rerio_
XP_015134812.2_LIM/homeobox_protein_Lhx2_isoform_X1__Gallus_gallus_
XP_006497796.1_LIM/homeobox_protein_Lhx2_isoform_X1__Mus_musculus_
NP_004780.3_LIM/homeobox_protein_Lhx2__Homo_sapiens_
NP_001032320.2_LIM/homeobox_protein_Lhx9_isoform_2__Danio_rerio_
XP_015145736.1_LIM/homeobox_protein_Lhx9_isoform_X1__Gallus_gallus_
NP_001036042.1_LIM/homeobox_protein_Lhx9_isoform_c__Mus_musculus_
NP_064589.2_LIM/homeobox_protein_Lhx9_isoform_1__Homo_sapiens_
XP_013794263.1_LIM/homeobox_protein_Lhx3_like_isoform_X2__Limulus_polyphemus_
NP_476606.1_Lim3__isoform_A__Drosophila_melanogaster_
XP_014791107.1_PREDICTED__LIM/homeobox_protein_Lhx3_like__partial__Octopus_bimaculoides
BAQ19230.1_Lhx3/4_LIM_homeobox_protein__partial__Lottia_gigantea_
NP_001116445.1_LIM/homeobox_protein_Lhx4__Danio_rerio_
XP_025009019.1_LIM/homeobox_protein_Lhx4_isoform_X1__Gallus_gallus_
NP_034842.2_LIM/homeobox_protein_Lhx4__Mus_musculus_
NP_203129.1_LIM/homeobox_protein_Lhx4__Homo_sapiens_
NP_001034742.1_LIM/homeobox_protein_Lhx3_isoform_a__Mus_musculus_
NP_835258.1_LIM/homeobox_protein_Lhx3_isoform_a__Homo_sapiens_
NP_571283.1_LIM/homeobox_protein_Lhx3__Danio_rerio_
NP_001025506.1_LIM/homeobox_protein_Lhx3__Gallus_gallus_
NP_571291.1_LIM/homeobox_protein_Lhx1__Danio_rerio_
NP_990744.1_LIM/homeobox_protein_Lhx1__Gallus_gallus_
NP_032524.1_LIM/homeobox_protein_Lhx1__Mus_musculus_
NP_005559.2_LIM/homeobox_protein_Lhx1__Homo_sapiens_
NP_571293.1_LIM/homeobox_protein_Lhx5__Danio_rerio_
NP_001307835.1_LIM/homeobox_protein_Lhx5__Gallus_gallus_
NP_032525.1_LIM/homeobox_protein_Lhx5__Mus_musculus_
XP_014778793.1_PREDICTED_Lhx1_like__Octopus_bimaculoides_
NP_071758.1_LIM/homeobox_protein_Lhx5__Homo_sapiens_
XP_022239095.1_LIM/homeobox_protein_Lhx5_like_isoform_X1__Limulus_polyphemus_
XP_011450364.1_PREDICTED__LIM/homeobox_protein_Lhx5__Crassostrea_gigas_
Doryteuthis_pealeii_Lhx1/5
XP_012946044.1_PREDICTED__LIM/homeobox_protein_Lhx5_like__partial__Aplysia_californica_
BAQ19229.1_Lhx1/5_LIM_homeobox_protein__partial__Lottia_gigantea_
ADG26732.1_Lhx1/5__partial__Platynereis_dumerilii_
ELU00131.1_hypothetical_protein_CAPTEDRAFT_168913__Capitella_teleta_
EFX66374.1_hypothetical_protein_DAPPUDRAFT_11288__partial__Daphnia_pulex_
NP_572505.1_LIM_homeobox_1__isoform_A__Drosophila_melanogaster_
XP_008194940.1_PREDICTED__LIM/homeobox_protein_Lhx5_isoform_X2__Tribolium_castaneum_
Lhx1/5
7.0

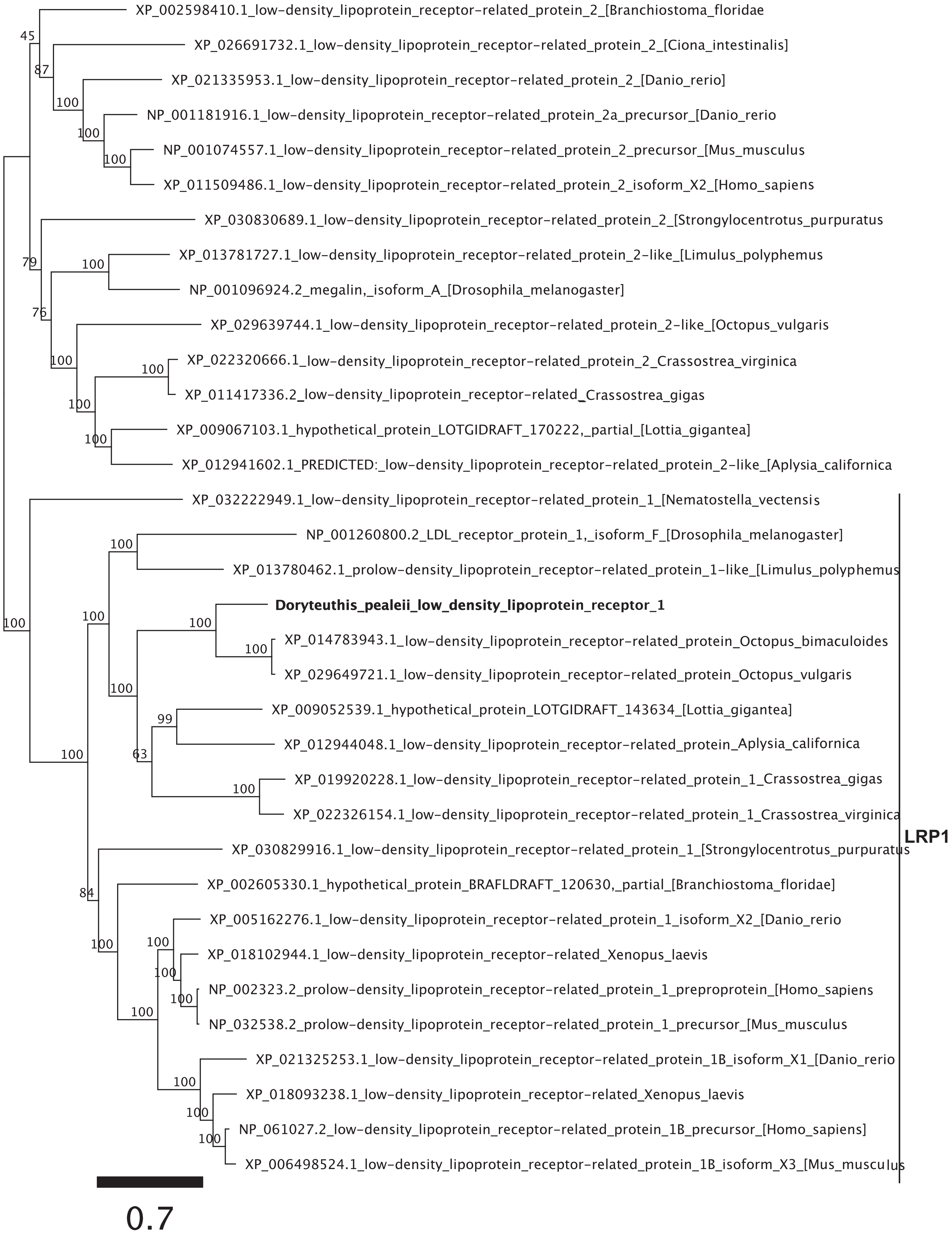

XP_002598410.1_low-density_lipoprotein_receptor-related_protein_2_[Branchiostoma_floridae
XP_026691732.1_low-density_lipoprotein_receptor-related_protein_2_[Ciona_intestinalis]
XP_021335953.1_low-density_lipoprotein_receptor-related_protein_2_[Danio_rerio]
NP_001181916.1_low-density_lipoprotein_receptor-related_protein_2a_precursor_[Danio_rerio
NP_001074557.1_low-density_lipoprotein_receptor-related_protein_2_precursor_[Mus_musculus
XP_011509486.1_low-density_lipoprotein_receptor-related_protein_2_isoform_X2_[Homo_sapiens
XP_030830689.1_low-density_lipoprotein_receptor-related_protein_2_[Strongylocentrotus_purpuratus
XP_013781727.1_low-density_lipoprotein_receptor-related_protein_2-like_[Limulus_polyphemus
NP_001096924.2_megalin,_isoform_A_[Drosophila_melanogaster]
XP_029639744.1_low-density_lipoprotein_receptor-related_protein_2-like_[Octopus_vulgaris
XP_022320666.1_low-density_lipoprotein_receptor-related_protein_2_Crassostrea_virginica
XP_011417336.2_low-density_lipoprotein_receptor-related_Crassostrea_gigas
XP_009067103.1_hypothetical_protein_LOTGIDRAFT_170222,_partial_[Lottia_gigantea]
XP_012941602.1_PREDICTED:_low-density_lipoprotein_receptor-related_protein_2-like_[Aplysia_californica
XP_032222949.1_low-density_lipoprotein_receptor-related_protein_1_[Nematostella_vectensis
NP_001260800.2_LDL_receptor_protein_1,_isoform_F_[Drosophila_melanogaster]
XP_013780462.1_prolow-density_lipoprotein_receptor-related_protein_1-like_[Limulus_polyphemus
Doryteuthis_pealeii_low_density_lipoprotein_receptor_1
XP_014783943.1_low-density_lipoprotein_receptor-related_protein_Octopus_bimaculoides
XP_029649721.1_low-density_lipoprotein_receptor-related_protein_Octopus_vulgaris
XP_009052539.1_hypothetical_protein_LOTGIDRAFT_143634_[Lottia_gigantea]
XP_012944048.1_low-density_lipoprotein_receptor-related_protein_Aplysia_californica
XP_019920228.1_low-density_lipoprotein_receptor-related_protein_1_Crassostrea_gigas
XP_022326154.1_low-density_lipoprotein_receptor-related_protein_1_Crassostrea_virginica
XP_030829916.1_low-density_lipoprotein_receptor-related_protein_1_[Strongylocentrotus_purpuratus
XP_002605330.1_hypothetical_protein_BRAFLDRAFT_120630,_partial_[Branchiostoma_floridae]
XP_005162276.1_low-density_lipoprotein_receptor-related_protein_1_isoform_X2_[Danio_rerio
XP_018102944.1_low-density_lipoprotein_receptor-related_Xenopus_laevis
NP_002323.2_prolow-density_lipoprotein_receptor-related_protein_1_preproprotein_[Homo_sapiens
NP_032538.2_prolow-density_lipoprotein_receptor-related_protein_1_precursor_[Mus_musculus
XP_021325253.1_low-density_lipoprotein_receptor-related_protein_1B_isoform_X1_[Danio_rerio
XP_018093238.1_low-density_lipoprotein_receptor-related_Xenopus_laevis
NP_061027.2_low-density_lipoprotein_receptor-related_protein_1B_precursor_[Homo_sapiens]
XP_006498524.1_low-density_lipoprotein_receptor-related_protein_1B_isoform_X3_[Mus_musculus
LRP1
0.7

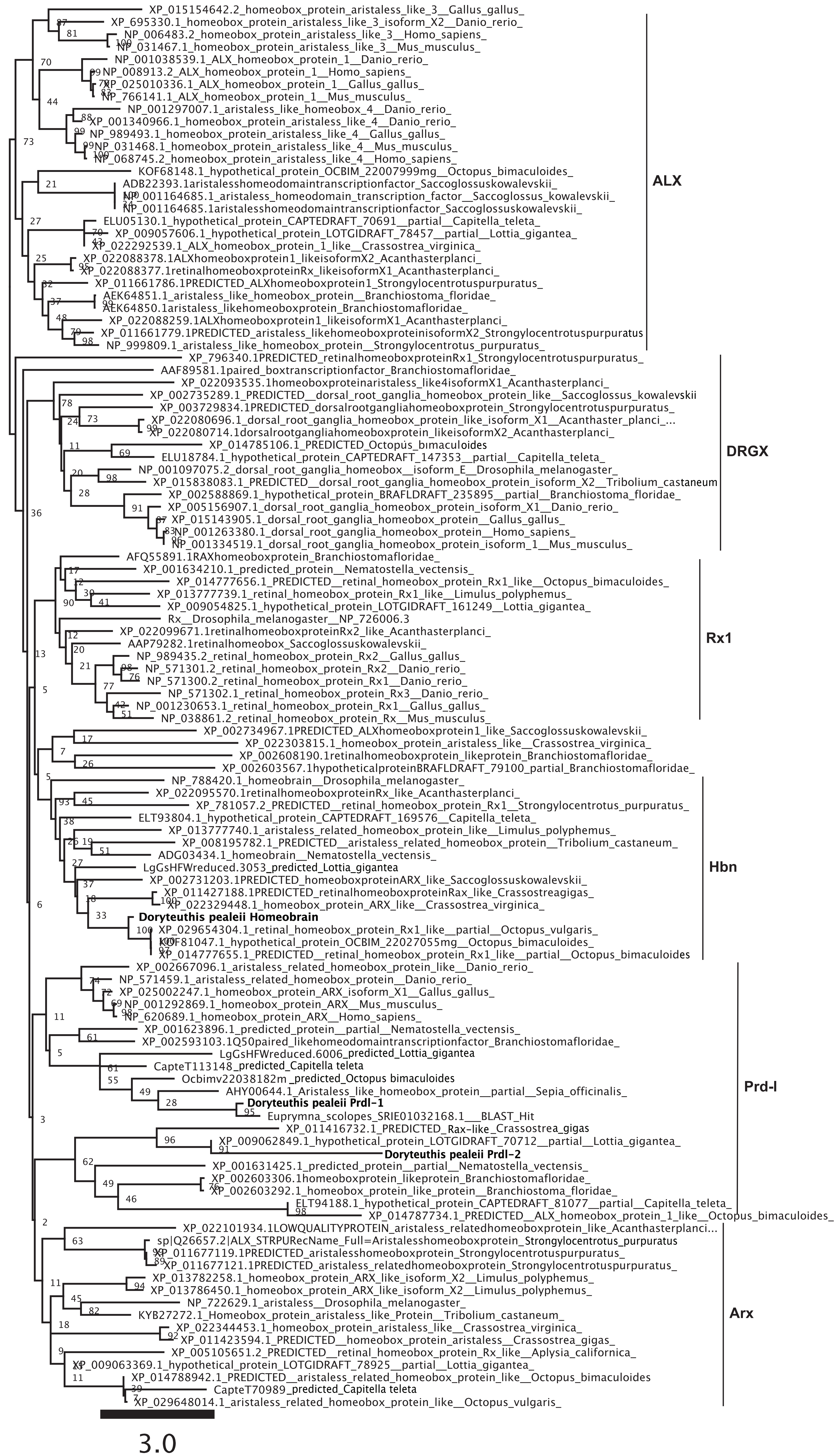
XP_015154642.2_homeobox_protein_aristaless_like_3__Gallus_gallus_
XP_695330.1_homeobox_protein_aristaless_like_3_isoform_X2__Danio_rerio_
NP_006483.2_homeobox_protein_aristaless_like_3__Homo_sapiens_
NP_031467.1_homeobox_protein_aristaless_like_3__Mus_musculus_
NP_001038539.1_ALX_homeobox_protein_1__Danio_rerio_
NP_008913.2_ALX_homeobox_protein_1__Homo_sapiens_
XP_025010336.1_ALX_homeobox_protein_1__Gallus_gallus_
NP_766141.1_ALX_homeobox_protein_1__Mus_musculus_
NP_001297007.1_aristaless_like_homeobox_4__Danio_rerio_
XP_001340966.1_homeobox_protein_aristaless_like_4__Danio_rerio_
NP_989493.1_homeobox_protein_aristaless_like_4__Gallus_gallus_
NP_031468.1_homeobox_protein_aristaless_like_4__Mus_musculus_
NP_068745.2_homeobox_protein_aristaless_like_4__Homo_sapiens_
KOF68148.1_hypothetical_protein_OCBIM_22007999mg__Octopus_bimaculoides_
ADB22393.1aristalesshomeodomaintranscriptionfactor_Saccoglossuskowalevskii_
NP_001164685.1_aristaless_homeodomain_transcription_factor__Saccoglossus_kowalevskii_
NP_001164685.1aristalesshomeodomaintranscriptionfactor_Saccoglossuskowalevskii_
ELU05130.1_hypothetical_protein_CAPTEDRAFT_70691__partial__Capitella_teleta_
XP_009057606.1_hypothetical_protein_LOTGIDRAFT_78457__partial__Lottia_gigantea_
XP_022292539.1_ALX_homeobox_protein_1_like__Crassostrea_virginica_
XP_022088378.1ALXhomeoboxprotein1_likeisoformX2_Acanthasterplanci_
XP_022088377.1retinalhomeoboxproteinRx_likeisoformX1_Acanthasterplanci_
XP_011661786.1PREDICTED_ALXhomeoboxprotein1_Strongylocentrotuspurpuratus_
AEK64851.1_aristaless_like_homeobox_protein__Branchiostoma_floridae_
AEK64850.1aristaless_likehomeoboxprotein_Branchiostomafloridae_
XP_022088259.1ALXhomeoboxprotein1_likeisoformX1_Acanthasterplanci_
XP_011661779.1PREDICTED_aristaless_likehomeoboxproteinisoformX2_Strongylocentrotuspurpuratus
NP_999809.1_aristaless_like_homeobox_protein__Strongylocentrotus_purpuratus_
ALX
XP_796340.1PREDICTED_retinalhomeoboxproteinRx1_Strongylocentrotuspurpuratus_
AAF89581.1paired_boxtranscriptionfactor_Branchiostomafloridae_
XP_022093535.1homeoboxproteinaristaless_like4isoformX1_Acanthasterplanci_
XP_002735289.1_PREDICTED__dorsal_root_ganglia_homeobox_protein_like__Saccoglossus_kowalevskii
XP_003729834.1PREDICTED_dorsalrootgangliahomeoboxprotein_Strongylocentrotuspurpuratus_
XP_022080696.1_dorsal_root_ganglia_homeobox_protein_like_isoform_X1__Acanthaster_planci_...
XP_022080714.1dorsalrootgangliahomeoboxprotein_likeisoformX2_Acanthasterplanci_
XP_014785106.1_PREDICTED_Octopus_bimaculoides
ELU18784.1_hypothetical_protein_CAPTEDRAFT_147353__partial__Capitella_teleta_
NP_001097075.2_dorsal_root_ganglia_homeobox__isoform_E__Drosophila_melanogaster_
XP_015838083.1_PREDICTED__dorsal_root_ganglia_homeobox_protein_isoform_X2__Tribolium_castaneum
XP_002588869.1_hypothetical_protein_BRAFLDRAFT_235895__partial__Branchiostoma_floridae_
XP_005156907.1_dorsal_root_ganglia_homeobox_protein_isoform_X1__Danio_rerio_
XP_015143905.1_dorsal_root_ganglia_homeobox_protein__Gallus_gallus_
NP_001263380.1_dorsal_root_ganglia_homeobox_protein__Homo_sapiens_
NP_001334519.1_dorsal_root_ganglia_homeobox_protein_isoform_1__Mus_musculus_
DRGX
AFQ55891.1RAXhomeoboxprotein_Branchiostomafloridae_
XP_001634210.1_predicted_protein__Nematostella_vectensis_
XP_014777656.1_PREDICTED__retinal_homeobox_protein_Rx1_like__Octopus_bimaculoides_
XP_013777739.1_retinal_homeobox_protein_Rx1_like__Limulus_polyphemus_
XP_009054825.1_hypothetical_protein_LOTGIDRAFT_161249__Lottia_gigantea_
Rx__Drosophila_melanogaster__NP_726006.3
XP_022099671.1retinalhomeoboxproteinRx2_like_Acanthasterplanci_
AAP79282.1retinalhomeobox_Saccoglossuskowalevskii_
NP_989435.2_retinal_homeobox_protein_Rx2__Gallus_gallus_
NP_571301.2_retinal_homeobox_protein_Rx2__Danio_rerio_
NP_571300.2_retinal_homeobox_protein_Rx1__Danio_rerio_
NP_571302.1_retinal_homeobox_protein_Rx3__Danio_rerio_
NP_001230653.1_retinal_homeobox_protein_Rx1__Gallus_gallus_
NP_038861.2_retinal_homeobox_protein_Rx__Mus_musculus_
Rx1
XP_002734967.1PREDICTED_ALXhomeoboxprotein1_like_Saccoglossuskowalevskii_
XP_022303815.1_homeobox_protein_aristaless_like__Crassostrea_virginica_
XP_002608190.1retinalhomeoboxprotein_likeprotein_Branchiostomafloridae_
XP_002603567.1hypotheticalproteinBRAFLDRAFT_79100_partial_Branchiostomafloridae_
NP_788420.1_homeobrain__Drosophila_melanogaster_
XP_022095570.1retinalhomeoboxproteinRx_like_Acanthasterplanci_
XP_781057.2_PREDICTED__retinal_homeobox_protein_Rx1__Strongylocentrotus_purpuratus_
ELT93804.1_hypothetical_protein_CAPTEDRAFT_169576__Capitella_teleta_
XP_013777740.1_aristaless_related_homeobox_protein_like__Limulus_polyphemus_
XP_008195782.1_PREDICTED__aristaless_related_homeobox_protein__Tribolium_castaneum_
ADG03434.1_homeobrain__Nematostella_vectensis_
LgGsHFWreduced.3053_predicted_Lottia_gigantea
XP_002731203.1PREDICTED_homeoboxproteinARX_like_Saccoglossuskowalevskii_
XP_011427188.1PREDICTED_retinalhomeoboxproteinRax_like_Crassostreagigas_
XP_022329448.1_homeobox_protein_ARX_like__Crassostrea_virginica_
Doryteuthis pealeii Homeobrain
XP_029654304.1_retinal_homeobox_protein_Rx1_like__partial__Octopus_vulgaris_
KOF81047.1_hypothetical_protein_OCBIM_22027055mg__Octopus_bimaculoides_
XP_014777655.1_PREDICTED__retinal_homeobox_protein_Rx1_like__partial__Octopus_bimaculoides
Hbn
XP_002667096.1_aristaless_related_homeobox_protein_like__Danio_rerio_
NP_571459.1_aristaless_related_homeobox_protein__Danio_rerio_
XP_025002247.1_homeobox_protein_ARX_isoform_X1__Gallus_gallus_
NP_001292869.1_homeobox_protein_ARX__Mus_musculus_
NP_620689.1_homeobox_protein_ARX__Homo_sapiens_
XP_001623896.1_predicted_protein__partial__Nematostella_vectensis_
XP_002593103.1Q50paired_likehomeodomaintranscriptionfactor_Branchiostomafloridae_
LgGsHFWreduced.6006_predicted_Lottia_gigantea
CapteT113148_predicted_Capitella teleta
Ocbimv22038182m _predicted_Octopus bimaculoides
AHY00644.1_Aristaless_like_homeobox_protein__partial__Sepia_officinalis_
Doryteuthis pealeii Prdl-1
Euprymna_scolopes_SRIE01032168.1___BLAST_Hit
XP_011416732.1_PREDICTED_Rax-like_Crassostrea_gigas
XP_009062849.1_hypothetical_protein_LOTGIDRAFT_70712__partial__Lottia_gigantea_
Doryteuthis pealeii Prdl-2
XP_001631425.1_predicted_protein__partial__Nematostella_vectensis_
XP_002603306.1homeoboxprotein_likeprotein_Branchiostomafloridae_
XP_002603292.1_homeobox_protein_like_protein__Branchiostoma_floridae_
ELT94188.1_hypothetical_protein_CAPTEDRAFT_81077__partial__Capitella_teleta_
XP_014787734.1_PREDICTED__ALX_homeobox_protein_1_like__Octopus_bimaculoides_
Prd-l
XP_022101934.1LOWQUALITYPROTEIN_aristaless_relatedhomeoboxprotein_like_Acanthasterplanci...
sp|Q26657.2|ALX_STRPURecName_Full=Aristaleshomeoboxprotein_Strongylocentrotus_purpuratus
XP_011677119.1PREDICTED_aristaleshomeoboxprotein_Strongylocentrotuspurpuratus_
XP_011677121.1PREDICTED_aristaless_relatedhomeoboxprotein_Strongylocentrotuspurpuratus_
XP_013782258.1_homeobox_protein_ARX_like_isoform_X2__Limulus_polyphemus_
XP_013786450.1_homeobox_protein_ARX_like_isoform_X2__Limulus_polyphemus_
NP_722629.1_aristaless__Drosophila_melanogaster_
KYB27272.1_Homeobox_protein_aristaless_like_Protein__Tribolium_castaneum_
XP_022344453.1_homeobox_protein_aristaless_like__Crassostrea_virginica_
XP_011423594.1_PREDICTED__homeobox_protein_aristaless__Crassostrea_gigas_
XP_005105651.2_PREDICTED__retinal_homeobox_protein_Rx_like__Aplysia_californica_
XP_009063369.1_hypothetical_protein_LOTGIDRAFT_78925__partial__Lottia_gigantea_
XP_014788942.1_PREDICTED__aristaless_related_homeobox_protein_like__Octopus_bimaculoides
CapteT70989_predicted_Capitella teleta
XP_029648014.1_aristaless_related_homeobox_protein_like__Octopus_vulgaris_
Arx
3.0

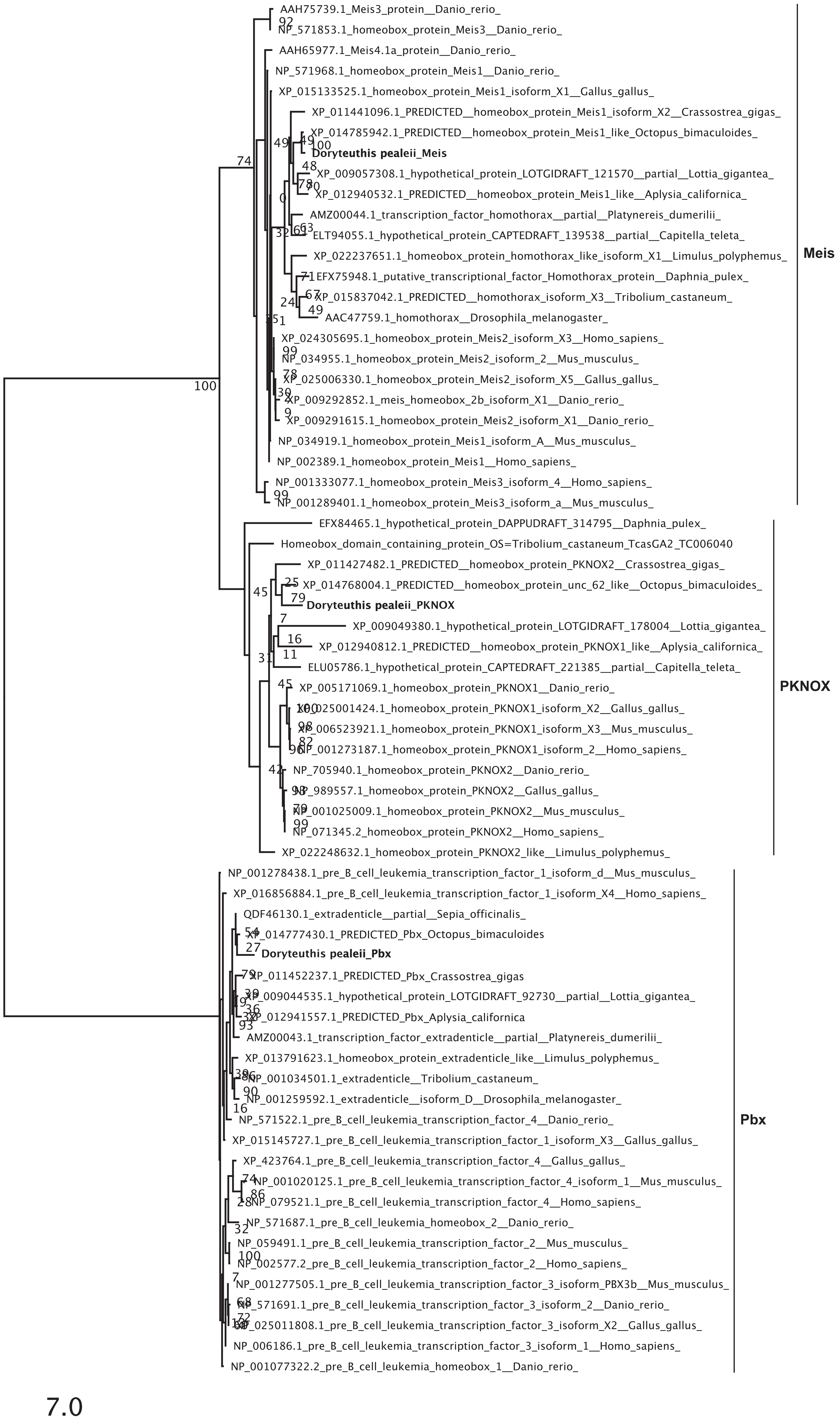
AAH75739.1_Meis3_protein__Danio_rerio_
NP_571853.1_homeobox_protein_Meis3__Danio_rerio_
AAH65977.1_Meis4.1a_protein__Danio_rerio_
NP_571968.1_homeobox_protein_Meis1__Danio_rerio_
XP_015133525.1_homeobox_protein_Meis1_isoform_X1__Gallus_gallus_
XP_011441096.1_PREDICTED__homeobox_protein_Meis1_isoform_X2__Crassostrea_gigas_
XP_014785942.1_PREDICTED__homeobox_protein_Meis1_like_Octopus_bimaculoides_
Doryteuthis pealeii_Meis
XP_009057308.1_hypothetical_protein_LOTGIDRAFT_121570__partial__Lottia_gigantea_
XP_012940532.1_PREDICTED__homeobox_protein_Meis1_like__Aplysia_californica_
AMZ00044.1_transcription_factor_homothorax__partial__Platynereis_dumerilii_
ELT94055.1_hypothetical_protein_CAPTEDRAFT_139538__partial__Capitella_teleta_
XP_022237651.1_homeobox_protein_homothorax_like_isoform_X1__Limulus_polyphemus_
EFX75948.1_putative_transcriptional_factor_Homothorax_protein__Daphnia_pulex_
XP_015837042.1_PREDICTED__homothorax_isoform_X3__Tribolium_castaneum_
AAC47759.1_homothorax__Drosophila_melanogaster_
XP_024305695.1_homeobox_protein_Meis2_isoform_X3__Homo_sapiens_
NP_034955.1_homeobox_protein_Meis2_isoform_2__Mus_musculus_
XP_025006330.1_homeobox_protein_Meis2_isoform_X5__Gallus_gallus_
XP_009292852.1_meis_homeobox_2b_isoform_X1__Danio_rerio_
XP_009291615.1_homeobox_protein_Meis2_isoform_X1__Danio_rerio_
NP_034919.1_homeobox_protein_Meis1_isoform_A__Mus_musculus_
NP_002389.1_homeobox_protein_Meis1__Homo_sapiens_
NP_001333077.1_homeobox_protein_Meis3_isoform_4__Homo_sapiens_
NP_001289401.1_homeobox_protein_Meis3_isoform_a__Mus_musculus_
Meis
EFX84465.1_hypothetical_protein_DAPPUDRAFT_314795__Daphnia_pulex_
Homeobox_domain_containing_protein_OS=Tribolium_castaneum_TcasGA2_TC006040
XP_011427482.1_PREDICTED__homeobox_protein_PKNOX2__Crassostrea_gigas_
XP_014768004.1_PREDICTED__homeobox_protein_unc_62_like__Octopus_bimaculoides_
Doryteuthis pealeii_PKNOX
XP_009049380.1_hypothetical_protein_LOTGIDRAFT_178004__Lottia_gigantea_
XP_012940812.1_PREDICTED__homeobox_protein_PKNOX1_like__Aplysia_californica_
ELU05786.1_hypothetical_protein_CAPTEDRAFT_221385__partial__Capitella_teleta_
XP_005171069.1_homeobox_protein_PKNOX1__Danio_rerio_
XP_025001424.1_homeobox_protein_PKNOX1_isoform_X2__Gallus_gallus_
XP_006523921.1_homeobox_protein_PKNOX1_isoform_X3__Mus_musculus_
NP_001273187.1_homeobox_protein_PKNOX1_isoform_2__Homo_sapiens_
NP_705940.1_homeobox_protein_PKNOX2__Danio_rerio_
NP_989557.1_homeobox_protein_PKNOX2__Gallus_gallus_
NP_001025009.1_homeobox_protein_PKNOX2__Mus_musculus_
NP_071345.2_homeobox_protein_PKNOX2__Homo_sapiens_
XP_022248632.1_homeobox_protein_PKNOX2_like__Limulus_polyphemus_
PKNOX
NP_001278438.1_pre_B_cell_leukemia_transcription_factor_1_isoform_d__Mus_musculus_
XP_016856884.1_pre_B_cell_leukemia_transcription_factor_1_isoform_X4__Homo_sapiens_
QDF46130.1_extradenticle__partial__Sepia_officinalis_
XP_014777430.1_PREDICTED_Pbx_Octopus_bimaculoides
Doryteuthis pealeii_Pbx
XP_011452237.1_PREDICTED_Pbx_Crassostrea_gigas
XP_009044535.1_hypothetical_protein_LOTGIDRAFT_92730__partial__Lottia_gigantea_
XP_012941557.1_PREDICTED_Pbx_Aplysia_californica
AMZ00043.1_transcription_factor_extradenticle__partial__Platynereis_dumerilii_
XP_013791623.1_homeobox_protein_extradenticle_like__Limulus_polyphemus_
NP_001034501.1_extradenticle__Tribolium_castaneum_
NP_001259592.1_extradenticle__isoform_D__Drosophila_melanogaster_
NP_571522.1_pre_B_cell_leukemia_transcription_factor_4__Danio_rerio_
XP_015145727.1_pre_B_cell_leukemia_transcription_factor_1_isoform_X3__Gallus_gallus_
XP_423764.1_pre_B_cell_leukemia_transcription_factor_4__Gallus_gallus_
NP_001020125.1_pre_B_cell_leukemia_transcription_factor_4_isoform_1__Mus_musculus_
NP_079521.1_pre_B_cell_leukemia_transcription_factor_4__Homo_sapiens_
NP_571687.1_pre_B_cell_leukemia_homeobox_2__Danio_rerio_
NP_059491.1_pre_B_cell_leukemia_transcription_factor_2__Mus_musculus_
NP_002577.2_pre_B_cell_leukemia_transcription_factor_2__Homo_sapiens_
NP_001277505.1_pre_B_cell_leukemia_transcription_factor_3_isoform_PBX3b__Mus_musculus_
NP_571691.1_pre_B_cell_leukemia_transcription_factor_3_isoform_2__Danio_rerio_
XP_025011808.1_pre_B_cell_leukemia_transcription_factor_3_isoform_X2__Gallus_gallus_
NP_006186.1_pre_B_cell_leukemia_transcription_factor_3_isoform_1__Homo_sapiens_
NP_001077322.2_pre_B_cell_leukemia_homeobox_1__Danio_rerio_
Pbx
7.0

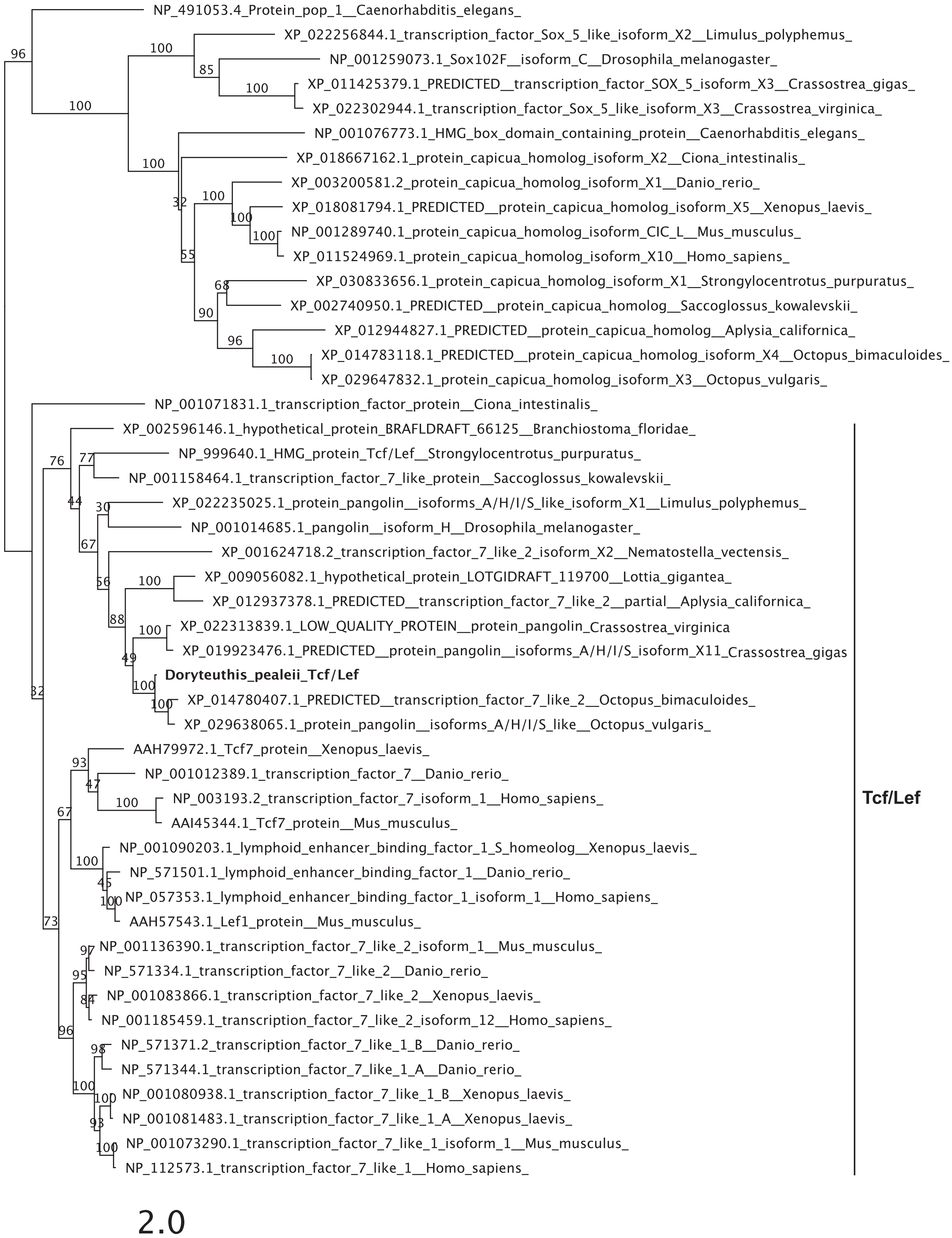
NP_491053.4_Protein_pop_1__Caenorhabditis_elegans_
XP_022256844.1_transcription_factor_Sox_5_like_isoform_X2__Limulus_polyphemus_
NP_001259073.1_Sox102F__isoform_C__Drosophila_melanogaster_
XP_011425379.1_PREDICTED__transcription_factor_SOX_5_isoform_X3__Crassostrea_gigas_
XP_022302944.1_transcription_factor_Sox_5_like_isoform_X3__Crassostrea_virginica_
NP_001076773.1_HMG_box_domain_containing_protein__Caenorhabditis_elegans_
XP_018667162.1_protein_capicua_homolog_isoform_X2__Ciona_intestinalis_
XP_003200581.2_protein_capicua_homolog_isoform_X1__Danio_rerio_
XP_018081794.1_PREDICTED__protein_capicua_homolog_isoform_X5__Xenopus_laevis_
NP_001289740.1_protein_capicua_homolog_isoform_CIC_L__Mus_musculus_
XP_011524969.1_protein_capicua_homolog_isoform_X10__Homo_sapiens_
XP_030833656.1_protein_capicua_homolog_isoform_X1__Strongylocentrotus_purpuratus_
XP_002740950.1_PREDICTED__protein_capicua_homolog__Saccoglossus_kowalevskii_
XP_012944827.1_PREDICTED__protein_capicua_homolog__Aplysia_californica_
XP_014783118.1_PREDICTED__protein_capicua_homolog_isoform_X4__Octopus_bimaculoides_
XP_029647832.1_protein_capicua_homolog_isoform_X3__Octopus_vulgaris_
NP_001071831.1_transcription_factor_protein__Ciona_intestinalis_
XP_002596146.1_hypothetical_protein_BRAFLDRAFT_66125__Branchiostoma_floridae_
NP_999640.1_HMG_protein_Tcf/Lef__Strongylocentrotus_purpuratus_
NP_001158464.1_transcription_factor_7_like_protein__Saccoglossus_kowalevskii_
XP_022235025.1_protein_pangolin__isoforms_A/H/I/S_like_isoform_X1__Limulus_polyphemus_
NP_001014685.1_pangolin__isoform_H__Drosophila_melanogaster_
XP_001624718.2_transcription_factor_7_like_2_isoform_X2__Nematostella_vectensis_
XP_009056082.1_hypothetical_protein_LOTGIDRAFT_119700__Lottia_gigantea_
XP_012937378.1_PREDICTED__transcription_factor_7_like_2__partial__Aplysia_californica_
XP_022313839.1_LOW_QUALITY_PROTEIN__protein_pangolin_Crassostrea_virginica
XP_019923476.1_PREDICTED__protein_pangolin__isoforms_A/H/I/S_isoform_X11_Crassostrea_gigas
Doryteuthis_pealeii_Tcf/Lef
XP_014780407.1_PREDICTED__transcription_factor_7_like_2__Octopus_bimaculoides_
XP_029638065.1_protein_pangolin__isoforms_A/H/I/S_like__Octopus_vulgaris_
AAH79972.1_Tcf7_protein__Xenopus_laevis_
NP_001012389.1_transcription_factor_7__Danio_rerio_
NP_003193.2_transcription_factor_7_isoform_1__Homo_sapiens_
AAI45344.1_Tcf7_protein__Mus_musculus_
NP_001090203.1_lymphoid_enhancer_binding_factor_1_S_homeolog__Xenopus_laevis_
NP_571501.1_lymphoid_enhancer_binding_factor_1__Danio_rerio_
NP_057353.1_lymphoid_enhancer_binding_factor_1_isoform_1__Homo_sapiens_
AAH57543.1_Lef1_protein__Mus_musculus_
NP_001136390.1_transcription_factor_7_like_2_isoform_1__Mus_musculus_
NP_571334.1_transcription_factor_7_like_2__Danio_rerio_
NP_001083866.1_transcription_factor_7_like_2__Xenopus_laevis_
NP_001185459.1_transcription_factor_7_like_2_isoform_12__Homo_sapiens_
NP_571371.2_transcription_factor_7_like_1_B__Danio_rerio_
NP_571344.1_transcription_factor_7_like_1_A__Danio_rerio_
NP_001080938.1_transcription_factor_7_like_1_B__Xenopus_laevis_
NP_001081483.1_transcription_factor_7_like_1_A__Xenopus_laevis_
NP_001073290.1_transcription_factor_7_like_1_isoform_1__Mus_musculus_
NP_112573.1_transcription_factor_7_like_1__Homo_sapiens_
Tcf/Lef
2.0

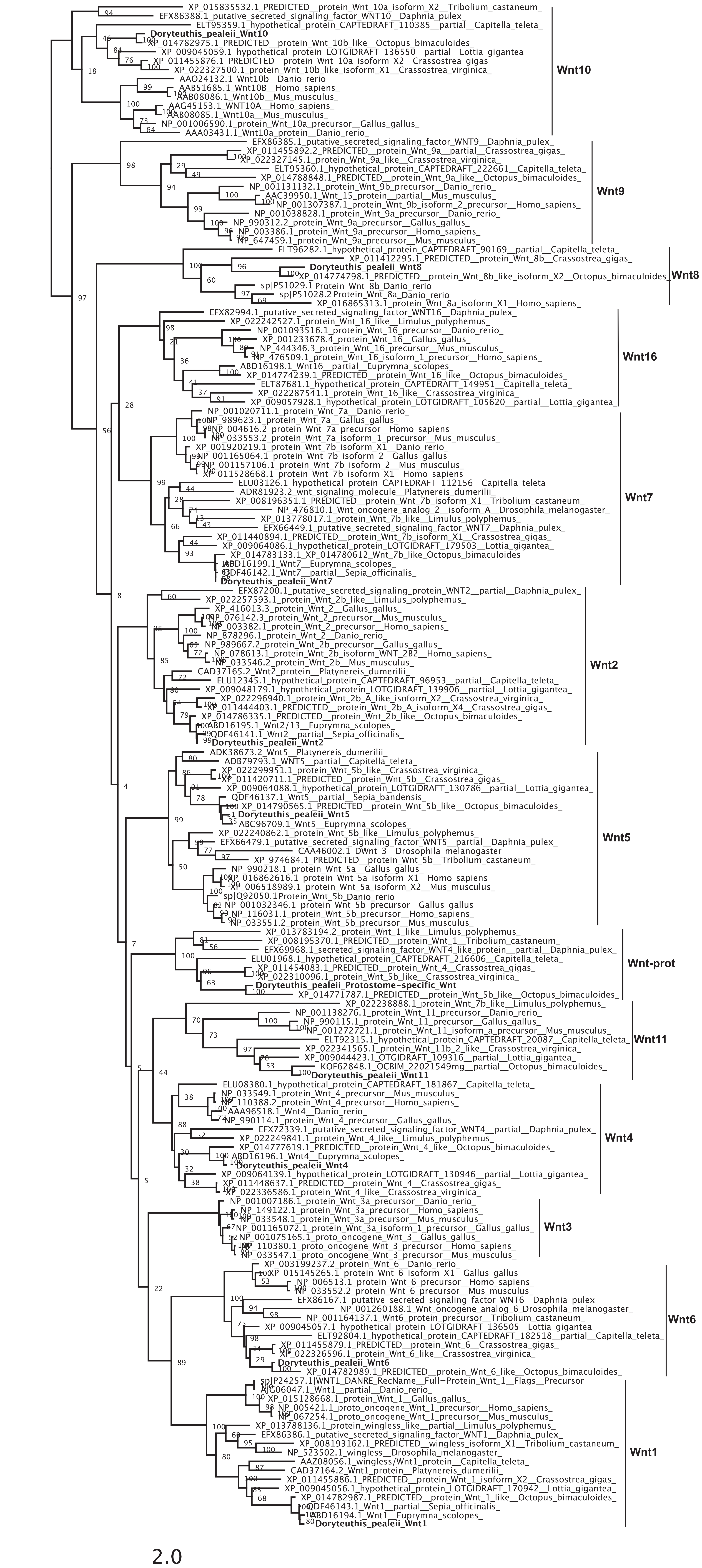
XP_015835532.1_PREDICTED__protein_Wnt_10a_isoform_X2__Tribolium_castaneum_
EFX86388.1_putative_secreted_signaling_factor_WNT10__Daphnia_pulex_
ELT95359.1_hypothetical_protein_CAPTEDRAFT_110385__partial__Capitella_teleta_
Doryteuthis_pealeii_Wnt10
XP_014782975.1_PREDICTED__protein_Wnt_10b_like__Octopus_bimaculoides_
XP_009045059.1_hypothetical_protein_LOTGIDRAFT_136550__partial__Lottia_gigantea_
XP_011455876.1_PREDICTED__protein_Wnt_10a_isoform_X2__Crassostrea_gigas_
XP_022327500.1_protein_Wnt_10b_like_isoform_X1__Crassostrea_virginica_
AAO24132.1_Wnt10b__Danio_rerio_
AAB51685.1_Wnt10B__Homo_sapiens_
AAB08086.1_Wnt10b__Mus_musculus_
AAG45153.1_WNT10A__Homo_sapiens_
AAB08085.1_Wnt10a__Mus_musculus_
NP_001006590.1_protein_Wnt_10a_precursor__Gallus_gallus_
AAA03431.1_Wnt10a_protein__Danio_rerio_
Wnt10
EFX86385.1_putative_secreted_signaling_factor_WNT9__Daphnia_pulex_
XP_011455892.2_PREDICTED__protein_Wnt_9a__partial__Crassostrea_gigas_
XP_022327145.1_protein_Wnt_9a_like__Crassostrea_virginica_
ELT95360.1_hypothetical_protein_CAPTEDRAFT_222661__Capitella_teleta_
XP_014788848.1_PREDICTED__protein_Wnt_9a_like__Octopus_bimaculoides_
NP_001131132.1_protein_Wnt_9b_precursor__Danio_rerio_
AAC39950.1_Wnt_15_protein__partial__Mus_musculus_
NP_001307387.1_protein_Wnt_9b_isoform_2_precursor__Homo_sapiens_
NP_001038828.1_protein_Wnt_9a_precursor__Danio_rerio_
NP_990312.2_protein_Wnt_9a_precursor__Gallus_gallus_
NP_003386.1_protein_Wnt_9a_precursor__Homo_sapiens_
NP_647459.1_protein_Wnt_9a_precursor__Mus_musculus_
Wnt9
ELT96282.1_hypothetical_protein_CAPTEDRAFT_90169__partial__Capitella_teleta_
XP_011412295.1_PREDICTED__protein_Wnt_8b__Crassostrea_gigas_
Doryteuthis_pealeii_Wnt8
XP_014774798.1_PREDICTED__protein_Wnt_8b_like_isoform_X2__Octopus_bimaculoides_
sp|P51029.1 Protein Wnt 8b_Danio_rerio
sp|P51028.2 Protein_Wnt_8a_Danio_rerio
XP_016865313.1_protein_Wnt_8a_isoform_X1__Homo_sapiens_
Wnt8
EFX82994.1_putative_secreted_signaling_factor_WNT16__Daphnia_pulex_
XP_022242527.1_protein_Wnt_16_like__Limulus_polyphemus_
NP_001093516.1_protein_Wnt_16_precursor__Danio_rerio_
XP_001233678.4_protein_Wnt_16__Gallus_gallus_
NP_444346.3_protein_Wnt_16_precursor__Mus_musculus_
NP_476509.1_protein_Wnt_16_isoform_1_precursor__Homo_sapiens_
ABD16198.1_Wnt16__partial__Euprymna_scolopes_
XP_014774239.1_PREDICTED__protein_Wnt_16_like__Octopus_bimaculoides_
ELT87681.1_hypothetical_protein_CAPTEDRAFT_149951__Capitella_teleta_
XP_022287541.1_protein_Wnt_16_like__Crassostrea_virginica_
XP_009057928.1_hypothetical_protein_LOTGIDRAFT_105620__partial__Lottia_gigantea_
Wnt16
NP_001020711.1_protein_Wnt_7a__Danio_rerio_
NP_989623.1_protein_Wnt_7a__Gallus_gallus_
NP_004616.2_protein_Wnt_7a_precursor__Homo_sapiens_
NP_033553.2_protein_Wnt_7a_isoform_1_precursor__Mus_musculus_
XP_001920219.1_protein_Wnt_7b_isoform_X1__Danio_rerio_
NP_001165064.1_protein_Wnt_7b_isoform_2__Gallus_gallus_
NP_001157106.1_protein_Wnt_7b_isoform_2__Mus_musculus_
XP_011528668.1_protein_Wnt_7b_isoform_X1__Homo_sapiens_
ELU03126.1_hypothetical_protein_CAPTEDRAFT_112156__Capitella_teleta_
ADR81923.2_wnt_signaling_molecule__Platynereis_dumerilii_
XP_008196351.1_PREDICTED__protein_Wnt_7b_isoform_X1__Tribolium_castaneum_
NP_476810.1_Wnt_oncogene_analog_2__isoform_A__Drosophila_melanogaster_
XP_013778017.1_protein_Wnt_7b_like__Limulus_polyphemus_
EFX66449.1_putative_secreted_signaling_factor_WNT7__Daphnia_pulex_
XP_011440894.1_PREDICTED__protein_Wnt_7b_isoform_X1__Crassostrea_gigas_
XP_009064086.1_hypothetical_protein_LOTGIDRAFT_179503__Lottia_gigantea_
XP_014783133.1_XP_014780612_Wnt_7b_like_Octopus_bimaculoides
ABD16199.1_Wnt7__Euprymna_scolopes_
QDF46142.1_Wnt7__partial__Sepia_officinalis_
Doryteuthis_pealeii_Wnt7
Wnt7
EFX87200.1_putative_secreted_signaling_protein_WNT2__partial__Daphnia_pulex_
XP_022257593.1_protein_Wnt_2b_like__Limulus_polyphemus_
XP_416013.3_protein_Wnt_2__Gallus_gallus_
NP_076142.3_protein_Wnt_2_precursor__Mus_musculus_
NP_003382.1_protein_Wnt_2_precursor__Homo_sapiens_
NP_878296.1_protein_Wnt_2__Danio_rerio_
NP_989667.2_protein_Wnt_2b_precursor__Gallus_gallus_
NP_078613.1_protein_Wnt_2b_isoform_WNT_2B2__Homo_sapiens_
NP_033546.2_protein_Wnt_2b__Mus_musculus_
CAD37165.2_Wnt2_protein__Platynereis_dumerilii_
ELU12345.1_hypothetical_protein_CAPTEDRAFT_96953__partial__Capitella_teleta_
XP_009048179.1_hypothetical_protein_LOTGIDRAFT_139906__partial__Lottia_gigantea_
XP_022296940.1_protein_Wnt_2b_A_like_isoform_X2__Crassostrea_virginica_
XP_011444403.1_PREDICTED__protein_Wnt_2b_A_isoform_X4__Crassostrea_gigas_
XP_014786335.1_PREDICTED__protein_Wnt_2b_like__Octopus_bimaculoides_
ABD16195.1_Wnt2/13__Euprymna_scolopes_
QDF46141.1_Wnt2__partial__Sepia_officinalis_
Doryteuthis_pealeii_Wnt2
Wnt2
ADK38673.2_Wnt5__Platynereis_dumerilii_
ADB79793.1_WNT5__partial__Capitella_teleta_
XP_022299951.1_protein_Wnt_5b_like__Crassostrea_virginica_
XP_011420711.1_PREDICTED__protein_Wnt_5b__Crassostrea_gigas_
XP_009064088.1_hypothetical_protein_LOTGIDRAFT_130786__partial__Lottia_gigantea_
QDF46137.1_Wnt5__partial__Sepia_bandensis_
XP_014790565.1_PREDICTED__protein_Wnt_5b_like__Octopus_bimaculoides_
Doryteuthis_pealeii_Wnt5
ABC96709.1_Wnt5__Euprymna_scolopes_
XP_022240862.1_protein_Wnt_5b_like__Limulus_polyphemus_
EFX66479.1_putative_secreted_signaling_factor_WNT5__partial__Daphnia_pulex_
CAA46002.1_DWnt_3__Drosophila_melanogaster_
XP_974684.1_PREDICTED__protein_Wnt_5b__Tribolium_castaneum_
NP_990218.1_protein_Wnt_5a__Gallus_gallus_
XP_016862616.1_protein_Wnt_5a_isoform_X1__Homo_sapiens_
XP_006518989.1_protein_Wnt_5a_isoform_X2__Mus_musculus_
sp|Q92050.1Protein_Wnt_5b_Danio_rerio
NP_001032346.1_protein_Wnt_5b_precursor__Gallus_gallus_
NP_116031.1_protein_Wnt_5b_precursor__Homo_sapiens_
NP_033551.2_protein_Wnt_5b_precursor__Mus_musculus_
Wnt5
XP_013783194.2_protein_Wnt_1_like__Limulus_polyphemus_
XP_008195370.1_PREDICTED__protein_Wnt_1__Tribolium_castaneum_
EFX69968.1_secreted_signaling_factor_WNT4_like_protein__partial__Daphnia_pulex_
ELU01968.1_hypothetical_protein_CAPTEDRAFT_216606__Capitella_teleta_
XP_011454083.1_PREDICTED__protein_Wnt_4__Crassostrea_gigas_
XP_022310096.1_protein_Wnt_5b_like__Crassostrea_virginica_
Doryteuthis_pealeii_Protostome-specific_Wnt
XP_014771787.1_PREDICTED__protein_Wnt_5b_like__Octopus_bimaculoides_
Wnt-prot
XP_022238888.1_protein_Wnt_7b_like__Limulus_polyphemus_
NP_001138276.1_protein_Wnt_11_precursor__Danio_rerio_
NP_990115.1_protein_Wnt_11_precursor__Gallus_gallus_
NP_001272721.1_protein_Wnt_11_isoform_a_precursor__Mus_musculus_
ELT92315.1_hypothetical_protein_CAPTEDRAFT_20087__Capitella_teleta_
XP_022341565.1_protein_Wnt_11b_2_like__Crassostrea_virginica_
XP_009044423.1_OTGIDRAFT_109316__partial__Lottia_gigantea_
KOF62848.1_OCBIM_22021549mg__partial__Octopus_bimaculoides_
Doryteuthis_pealeii_Wnt11
Wnt11
ELU08380.1_hypothetical_protein_CAPTEDRAFT_181867__Capitella_teleta_
NP_033549.1_protein_Wnt_4_precursor__Mus_musculus_
NP_110388.2_protein_Wnt_4_precursor__Homo_sapiens_
AAA96518.1_Wnt4__Danio_rerio_
NP_990114.1_protein_Wnt_4_precursor__Gallus_gallus_
EFX72339.1_putative_secreted_signaling_factor_WNT4__partial__Daphnia_pulex_
XP_022249841.1_protein_Wnt_4_like__Limulus_polyphemus_
XP_014777619.1_PREDICTED__protein_Wnt_4_like__Octopus_bimaculoides_
ABD16196.1_Wnt4__Euprymna_scolopes_
Doryteuthis_pealeii_Wnt4
XP_009064139.1_hypothetical_protein_LOTGIDRAFT_130946__partial__Lottia_gigantea_
XP_011448637.1_PREDICTED__protein_Wnt_4__Crassostrea_gigas_
XP_022336586.1_protein_Wnt_4_like__Crassostrea_virginica_
Wnt4
NP_001007186.1_protein_Wnt_3a_precursor__Danio_rerio_
NP_149122.1_protein_Wnt_3a_precursor__Homo_sapiens_
NP_033548.1_protein_Wnt_3a_precursor__Mus_musculus_
NP_001165072.1_protein_Wnt_3a_isoform_1_precursor__Gallus_gallus_
NP_001075165.1_proto_oncogene_Wnt_3__Gallus_gallus_
NP_110380.1_proto_oncogene_Wnt_3_precursor__Homo_sapiens_
NP_033547.1_proto_oncogene_Wnt_3_precursor__Mus_musculus_
Wnt3
XP_003199237.2_protein_Wnt_6__Danio_rerio_
XP_015145265.1_protein_Wnt_6_isoform_X1__Gallus_gallus_
NP_006513.1_protein_Wnt_6_precursor__Homo_sapiens_
NP_033552.2_protein_Wnt_6_precursor__Mus_musculus_
EFX86167.1_putative_secreted_signaling_factor_WNT6__Daphnia_pulex_
NP_001260188.1_Wnt_oncogene_analog_6_Drosophila_melanogaster_
NP_001164137.1_Wnt6_protein_precursor__Tribolium_castaneum_
XP_009045057.1_hypothetical_protein_LOTGIDRAFT_136505__Lottia_gigantea_
ELT92804.1_hypothetical_protein_CAPTEDRAFT_182518__partial__Capitella_teleta_
XP_011455879.1_PREDICTED__protein_Wnt_6__Crassostrea_gigas_
XP_022326596.1_protein_Wnt_6_like__Crassostrea_virginica_
Doryteuthis_pealeii_Wnt6
XP_014782989.1_PREDICTED__protein_Wnt_6_like__Octopus_bimaculoides_
Wnt6
sp|P24257.1|WNT1_DANRE_RecName__Full=Protein_Wnt_1__Flags__Precursor
AJG06047.1_Wnt1__partial__Danio_rerio_
XP_015128668.1_protein_Wnt_1__Gallus_gallus_
NP_005421.1_proto_oncogene_Wnt_1_precursor__Homo_sapiens_
NP_067254.1_proto_oncogene_Wnt_1_precursor__Mus_musculus_
XP_013788136.1_protein_wingless_like__partial__Limulus_polyphemus_
EFX86386.1_putative_secreted_signaling_factor_WNT1__Daphnia_pulex_
XP_008193162.1_PREDICTED__wingless_isoform_X1__Tribolium_castaneum_
NP_523502.1_wingless__Drosophila_melanogaster_
AAZ08056.1_wingless/Wnt1_protein__Capitella_teleta_
CAD37164.2_Wnt1_protein__Platynereis_dumerilii_
XP_011455886.1_PREDICTED__protein_Wnt_1_isoform_X2__Crassostrea_gigas_
XP_009045056.1_hypothetical_protein_LOTGIDRAFT_170942__Lottia_gigantea_
XP_014782987.1_PREDICTED__protein_Wnt_1_like__Octopus_bimaculoides_
QDF46143.1_Wnt1__partial__Sepia_officinalis_
ABD16194.1_Wnt1__Euprymna_scolopes_
Doryteuthis_pealeii_Wnt1
Wnt1
2.0

Fig. S2. Limb network supplemental data.

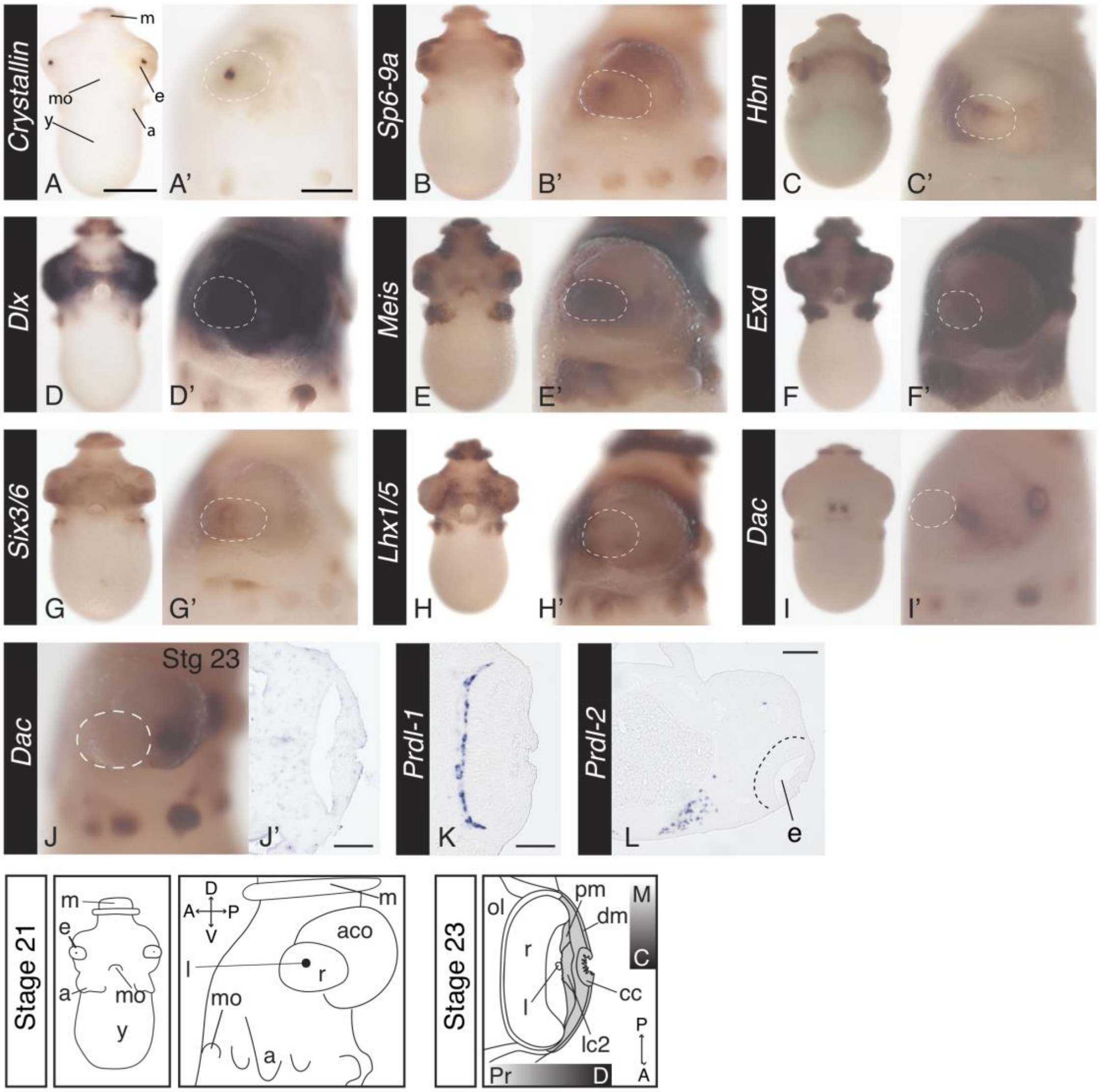

Fig. S2. Limb network supplemental data. A-I) Gene expression at stage 21 for limb network genes. For all genes from left to right, Anterior whole-mount and lateral whole-mount, anterior to the left. Scale for whole-mount anterior view is 500 microns. Scale for lateral whole-mount view 200 microns. J, J') Stage 23 Dac expression. J) Lateral whole mount, anterior to the left. J') Sectioned image of the eye. Anterior is down. K & L) Sectioned image of expression of Prdl-1and Prdl-2. Scale is 50 microns on eye sections, 100 microns on brain section (Prdl-2). *a*, arms; *aco*, anterior chamber organ; *cc*, cup cells; *dm,* distal-marginal cells; *e*, eye; *l*, lens; *lc2,* LC2 cells; *m*, mantle; *mo*, mouth; *pm,* proximal-marginal cells; r, retina; *y*, yolk. Anterior segment highlighted in grey in the cartoon. Orientation abbreviations: M, marginal; C, central; Pr, proximal; D, Distal; A, anterior; P, posterior.

Fig. S3. Targeted image enlargements of anterior segment gene expression

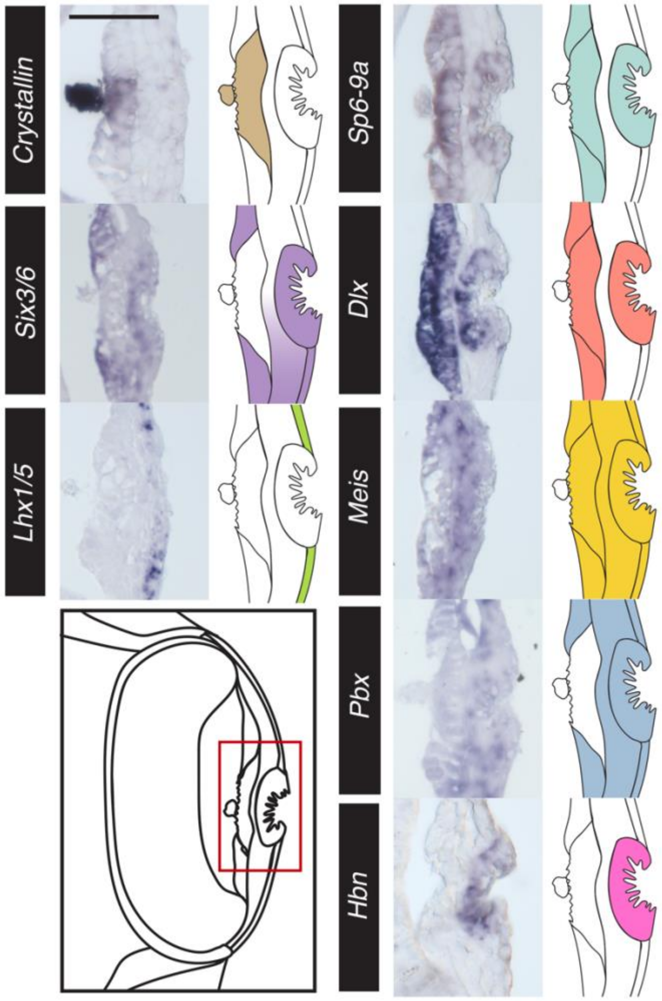

Fig. S3. Targeted image enlargements of anterior segment gene expression
Enlargements of central eye sections. Enlarged area shown by the red rectangle on the eye schematic. Cropped versions of the schematic summaries shown in Figure 2 are next to the corresponding gene expression. Scale is 50 microns.

Fig. S4. Wnt signaling expression supplemental data

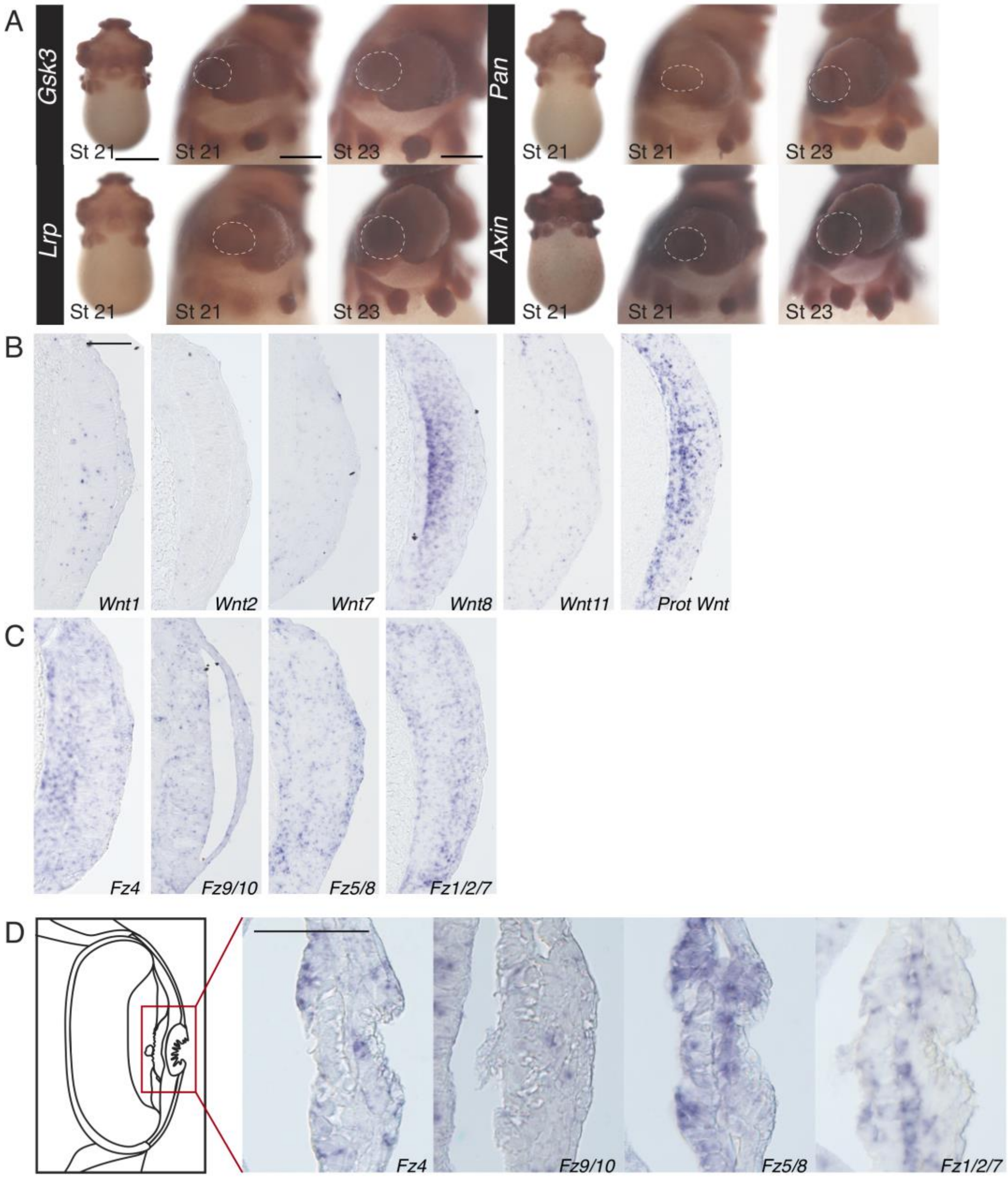

Fig. S4. Wnt signaling expression supplemental data. A) Wnt signaling pathway member expression, *Gsk3, Lrp, Pan*, and *Axin*, at stage 21 and 23 in whole-mount. Anterior view of stage 21and lateral views of stage 21 and stage 23 (anterior to the left). B) Wnt gene expression at stage 21 in section. Anterior is down. C) Fz receptor gene expression at stage 21. Anterior is down. D) Enlargements of stage 23 Fz expression as shown in Figure 3. Scale for whole-mount anterior view is 500 microns. Scale for lateral whole-mount view 200 microns. Scale for sectioned images 50 microns.

Fig. S5. Wnt agonist and antagonist supplemental data

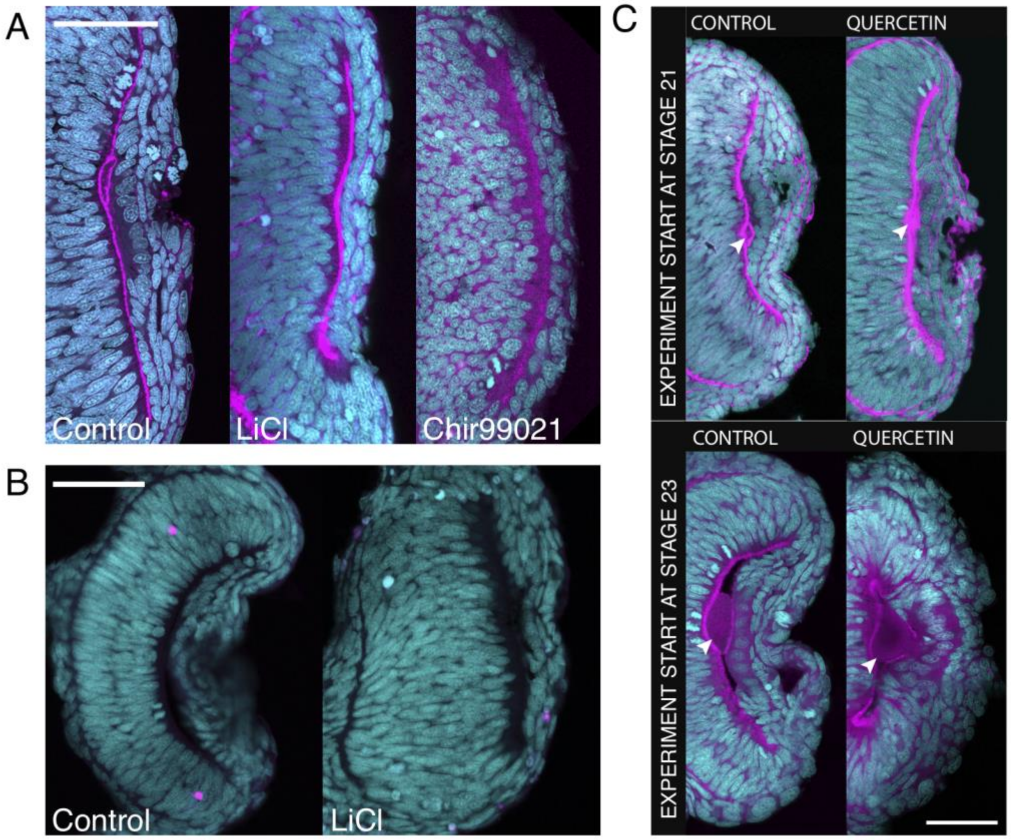

Fig. S5. Wnt agonist and antagonist supplemental data. A) Wnt agonist experiments starting at stage 21. Embryos were treated for 24 hours and fixed immediately. LiCl and Chir99021 show similar phenotypes: Lack of anterior segment thickness and loss of lens formation. Sytox nuclear stain in cyan, Phalloidin stain in magenta. Scale is 50 microns. B) Tunel staining of the eye of Control and LiCl treated embryos. Sytox nuclear stain in cyan, Tunel stain in magenta. Similar amounts of cell death observed in control and treated animals. Scale is 100 microns C) Wnt antagonist experiments starting at stage 21 and stage 23. 50uM Quercetin and DMSO control. Embryos were treated for 24 hours and fixed immediately. Arrowhead identifies developing lens. Scale is 50 microns.

Fig. S6 Supplemental in situ hybridization data and quantification

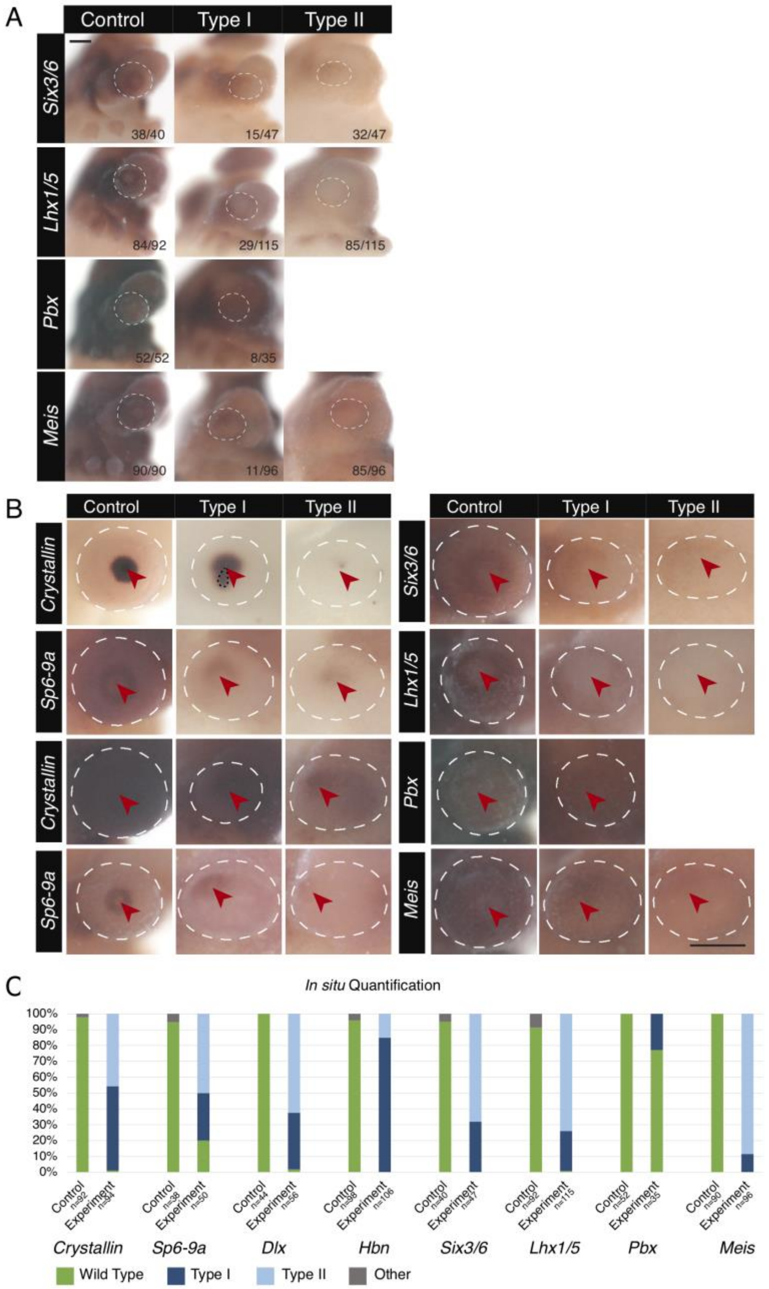

Fig. S6 Supplemental in situ hybridization data and quantification. A) In situ hybridization of limb patterning program members and and anterior segment markers after LiCl treatment. Type I (mild) and Type II (severe) phenotype. White dotted line outlines the eye in the lateral image. Number of eyes scored in control and the two phenotypes found in LiCl treated animals in the bottom right corner. Scale for lateral whole-mount view 200 microns. B) Enlargements of LiCl in situ hybridizations. Arrowhead identifies the site of normal lens formation. Black dotted line in *DpCrystalin* expression identifies the lens rudiment. This is to highlight the dorsal lentigenic cell expression, lens rudiment expression, and the lack of ventral expression. Scale 200 microns. C) Quantification of in situ hybridization experimental data shown in the corners of each image in Figure 3S-P'' and Supplemental Figure 6A.

Table S1. All Primer sequences

	Forward	Reverse
DpS-Crystallin	GAACATCATGTCGCACCACG	AGTTGCTCGCCTGAGAAGAC
DpLhx1/5	GAAGTGTCTTCGTGCTCCCA	ATTATCGACCGGCGAGGAAC
DpDlx	GGCAAGGCTTGGGTAAAACG	GGGGTAGCAGCGATGAGTTT
DpMeis	TAGCGTTTCCAAAAGGACCT	CCCCAATACCCGTCATACTC
DpPbx	TACTTCGGGAGCAGAGTCGA	TAGCGGTCGTCGTCGTAATG
DpHbn	ATACAACGACGACGACCACC	CGCGTGAATACATCCGGGTA
DpPrdl-1	AGAACAACCCAACGTACACA	GCAAACATCGAGTGAATCCC
DpPrdl-2	TCGCATTGAGGATTGATCTT	GGTTGTTGTTGTTGTGTTGTT
DpDac	CTGTATGGCTCCAAGTCCTC	GATCTCTGGTCGTCGTTTCA
DpGSK3	GATACGGGTGAACTGGTAGCAATC	CACCAACTGGATAGCCTCTGATG
DpLRP1	TTCCTTGAATAGCCTCATCGGTC	TTCCAAAAAGTGGGTGTGCG
DpAxin	CCCTCATTATTCTCCAACCTCCTC	CACAGAGCACTTCAAAAACGGG
DpTCF/LEF	GCTTGGGTGGCAAAATGTCG	TGCTGGACTGTTCTGGCAAAC
DpDvl	GCAGGCACTTTTTTTAGTAGCGTG	ATGTCCGTTGATGCGAGGTG
DpWnt-Prot	GACAGCCTACCTTTATGCCA	TACATTCGCAGTCTTCCGTT
DpWnt1	GTTTGCTTGTATTCGTGCGA	CCCTCCAATCCCAATGAAGT
DpWnt2	GTCGTTTGTGGTCCTTGTTG	GAATGTCAGTTCCAGTTGCG
DpWnt7	GTGCGTTGATGAATCTCCAC	TGTACTCCTCCGTCTTGTTG
DpWnt8	CTGCCAGATACTCCGTGACATTTAC	TTGGTTGGGGAATCGCACTG
DpWnt11	CTTGACATAGCAGCACCACACG	GAACAGTTTGCCAACAGAAGATGG
DpFz9/10	CGTAGTTTCTTGCCCGTAGAC	CGCTGTTTTGTATCAACCCCA
DpFz1/2/7	AAAGCCCCTTAAAGCATCCA	GACCATGCAATTCCACCTTG
DpFz4	TCAGTTCGTCAGCATCAACAT	CCGATATCCTCAACTGCACAA
DpFz5/8	TATTTGCTACCCACGGATCGC	CCGACCACCAAACACATAAAGT
